# *Candida albicans* Pma1p Contributes to Growth, pH Homeostasis, and Hyphal Formation

**DOI:** 10.3389/fmicb.2019.01012

**Published:** 2019-05-09

**Authors:** Hallie S. Rane, Summer R. Hayek, Jillian E. Frye, Esteban L. Abeyta, Stella M. Bernardo, Karlett J. Parra, Samuel A. Lee

**Affiliations:** ^1^Department of Biochemistry and Molecular Biology, University of New Mexico Health Science Center, Albuquerque, NM, United States; ^2^Section of Infectious Diseases, New Mexico VA Healthcare System, Albuquerque, NM, United States; ^3^Division of Infectious Diseases, University of New Mexico Health Science Center, Albuquerque, NM, United States; ^4^Medicine Service, White River Junction VA Medical Center, White River Junction, VT, United States; ^5^Infectious Disease Section, Department of Medicine, Geisel School of Medicine at Dartmouth, Dartmouth College, Hanover, NH, United States

**Keywords:** *Candida albicans*, cytosolic pH, filamentation, glucose metabolism, pH homeostasis, plasma membrane H^+^ ATPase

## Abstract

*Candida albicans* occupies diverse ecological niches within the host and must tolerate a wide range of environmental pH. The plasma membrane H^+^-ATPase Pma1p is the major regulator of cytosolic pH in fungi. Pma1p extrudes protons from the cytosol to maintain neutral-to-alkaline pH and is a potential drug target due to its essentiality and fungal specificity. We characterized mutants in which one allele of *PMA1* has been deleted and the other truncated by 18–38 amino acids. Increasing C-terminal truncation caused corresponding decreases in plasma membrane ATPase-specific activity and cytosolic pH. Pma1p is regulated by glucose: glucose rapidly activates the ATPase, causing a sharp increase in cytosolic pH. Increasing Pma1p truncation severely impaired this glucose response. Pma1p truncation also altered cation responses, disrupted vacuolar morphology and pH, and reduced filamentation competence. Early studies of cytosolic pH and filamentation have described a rapid, transient alkalinization of the cytosol preceding germ tube formation; Pma1p has been proposed as a regulator of this process. We find Pma1p plays a role in the establishment of cell polarity, and distribution of Pma1p is non-homogenous in emerging hyphae. These findings suggest a role of *PMA1* in cytosolic alkalinization and in the specialized form of polarized growth that is filamentation.

## Introduction

The opportunistic yeast *Candida albicans* colonizes an array of ecological niches in the human host, from the oral cavity to the stomach, lower gastrointestinal tract, and the genito-urinary tract, and during infection has the ability to invade the bloodstream and various organs. Survival in such diverse microenvironments requires an ability to adapt to an unusually broad range of external pH. *C. albicans* can survive in both extremely acidic (pH < 2) and extremely alkaline (pH > 10) environments. One component of this adaptability is the ability to regulate cytosolic pH. Unlike non-pathogenic yeasts such as *Saccharomyces cerevisiae*, which maintains a cytosolic pH between 6.0 and 7.0, cytosolic pH in *C. albicans* is both more alkaline and more tractable, with cytosolic pH values *in vivo* ranging between pH 5.8 and pH 9 (Cassone et al., 1983; Kaur et al., 1988; Stewart et al., 1988, 1989; Rabaste et al., 1995; Liu and Köhler, 2015; Tournu et al., 2017). Therefore, the study of cytosolic pH homeostasis and its role in both external pH adaptation and virulence is of interest in this versatile opportunistic pathogen.

The plasma membrane H^+^-ATPase Pma1p is the major regulator of cytosolic pH in plants and fungi. Pma1p hydrolyzes ATP to power the extrusion of protons from the cytosol. This functions to maintain neutral-to-alkaline pH, maintain ion balance, and drive nutrient uptake via generation of an electrochemical gradient. Although absent in mammals, Pma1p is distantly related to other P-type ATPase pumps including mammalian Na^+^, K^+^, and Ca^2+^ ATPases (Serrano et al., 1986; Ambesi et al., 2000). Pma1p is an integral component of the cell and is essential for growth. The *PMA1* gene is so highly and constitutively expressed that it is commonly used as a reference gene in RNA quantification studies (Nailis et al., 2006). The Pma1p protein is a major structural component of the plasma membrane, making up 20–40% of total plasma membrane protein in *C. albicans* (Monk et al., 1991). The H^+^-ATPase consumes an impressive percentage of cellular ATP, responsible for up to one quarter of all ATP consumption (Rao and Slayman, 1996). These traits highlight the biological importance of Pma1p as well as the potential utility of Pma1p as an antifungal target. Indeed, Pma1p has been validated as an antifungal drug target, although clinically useful inhibitors of Pma1p have not yet been identified (Stewart et al., 1988; Monk et al., 1995b, 2005; Perlin et al., 1997; Chan et al., 2007; Billack et al., 2009; Ottilie et al., 2018).

Previous work has shown that dramatic cytosolic alkalinization precedes hyphal formation in *C. albicans*; Pma1p has been proposed as a possible mediator of this phenomenon (Stewart et al., 1988). This hypothesis has not been confirmed, although there is indirect supporting evidence: emerging germ tubes have twice the level of plasma membrane ATPase-specific activity as budding cells, and treatment of cells with proton pump inhibitors blocks both ATPase-specific activity and morphogenesis (Kaur et al., 1988; Stewart et al., 1988, 1989; Monk et al., 1993). *PMA1* was also included in a large-scale virulence study using a mouse model of disseminated candidiasis wherein genes were placed under the control of a tet promoter system to analyze the role of essential genes to *in vivo* virulence (Becker et al., 2010). In this system, repression of *PMA1* completely abolished *C. albicans* virulence (Becker et al., 2010). Although the essentiality of Pma1p to *C. albicans* growth and virulence has been previously established (Monk et al., 1995a; Segal et al., 2018), it is still unknown whether Pma1p plays a direct or indirect role in pathogenesis. To date, no detailed genetic studies of Pma1p have been conducted in *C. albicans*, likely due to the essential nature of the gene. We therefore set out to define the roles of Pma1p and cytosolic pH homeostasis in *C. albicans* pathogenesis.

In *S. cerevisiae*, *PMA1* has been characterized through the study of partial loss-of-function mutants (McCusker et al., 1987; Portillo and Serrano, 1988; Portillo et al., 1989, 1991; Ambesi et al., 2000; Petrov, 2010; Mason et al., 2014). Mutations in *PMA1* have pleiotropic effects, including increased susceptibility to low extracellular pH, weak acids, osmotic pressure, and low temperature (McCusker et al., 1987). Differences in budding patterns, cell sizes, and cytokinesis have also been observed (McCusker et al., 1987). In *S. cerevisiae*, the N-terminus of Pma1 is involved in membrane insertion, whereas the C-terminus is involved in both membrane insertion and in the response to glucose (Portillo et al., 1989). One genetic method to study the function of *PMA1* involves generating C-terminal truncation mutants, since this domain is required for protein stability and proper trafficking to the plasma membrane. In *S. cerevisiae* such truncations support partial cell growth despite decreased levels of Pma1p activity as well as differences in structure and function of the plasma membrane H^+^-ATPase enzyme (Mason et al., 2014). *C. albicans* has a single homolog that is 83% similar to *S. cerevisiae* Pma1p (Monk et al., 1991). In this study, we created a series of *C. albicans* Pma1p truncation mutants in which one allele of *PMA1* was deleted and the second truncated by 18, 30, 32, 34, and 38 amino acids, respectively. C-terminal truncation of the Pma1p protein decreased plasma membrane ATPase-specific activity by 44.1–78.2%. We used these partial loss-of-function mutants to assess the role of *PMA1* in *C. albicans* cell biology, hyphal formation, and virulence.

## Materials and Methods

### Strains and Media

Strains used in this study are listed in Supplemental Table 1. Standard growth was completed at 30°C in unbuffered yeast peptone dextrose (YPD, 1% yeast extract, 2% peptone, and 2% glucose, pH ∼6.8) supplemented with 80 μg/mL uridine where required. For the tetR-*PMA1* strain, doxycycline was added when needed to a final concentration of 20 μg/mL. Calibration buffers for pHLuorin measurements were composed of 50 mM MES (2-(N-morpholino)ethanesulfonic acid), 50 mM HEPES (4-(2-hydroxyethyl)-1-piperazineethanesulfonic acid), 50 mM KCl, 50 mM NaCl, 0.2 M ammonium acetate, and 10 mM 2-deoxyglucose supplemented with 55 μM monensin, an ionophore. Previous studies have used calibration buffers prepared as described here, but with the addition of the preservative sodium azide and the ionophore nigericin (Diakov et al., 2013). We tested calibration buffers without these toxic ingredients and were able to obtain accurate, precise and replicable pH measurements; thus, these two ingredients were omitted as a safety precaution. Media was buffered to pH 4.0–5.0 using 50 mM succinic acid/50 mM Na_2_PO_4_ or to pH 7.5–8.5 using 50 mM MES hydrate/50 mM MOPS where required. Unless otherwise specified, agar plates were prepared with 2% agar. Where the pH of the media is not explicitly stated, the media used was not buffered to a specific pH (i.e., unbuffered media). All genomic DNA extractions were performed as described previously (Bernardo and Lee, 2010). All strains were constructed via lithium acetate transformation.

### Construction of tetR-*PMA1* and *pmp1Δ/Δ* Strains

To create a strain in which *PMA1* expression was under the control of a tetracycline-regulatable promoter, we deleted one *C. albicans PMA1* allele in the THE1 strain background using PCR-based gene disruption (Wilson et al., 2000). The *dpl200-URA3-dpl200*-containing plasmid pDDB57 was amplified with primers *PMA1*-5DR and *PMA1*-3DR (Supplemental Table 2). Strain THE1 was transformed with the resulting PCR amplicon to generate THE1-*PMA1*Δ*/+*. Correct genomic integration of the gene disruption cassette was confirmed via PCR using primers *PMA1*-5Det and *PMA1*-3Det. The THE1-*PMA1*Δ/+ strain was plated to FOA agar media and the resultant FOA-resistant colonies were screened via PCR for the *PMA1/PMA1*Δ::*dpl200* genotype using primers *PMA1*-5Det and *PMA1*-3Det. The tetracycline-regulatable system described by Nakayama et al. (2000) with modifications described by Bates et al. (2006) was used to place the remaining *PMA1* allele under a tetracycline-regulatable promoter. Plasmid p99CAU1 (from H. Nakayama, Suzuka University) was amplified using primers tet*PMA1*-5DR and tet*PMA1*-3DR. The resulting PCR amplicon was inserted upstream of the remaining *PMA1* allele of the THE1-*PMA1*Δ/+ FOA strain. Transformants were screened for correct insertion of the tetR-*PMA1* allele using primers *PMA1*-5Det and tet*PMA1*-3Det (Supplemental Table 2).

A PCR-based gene disruption strategy was also used to disrupt the potential plasma membrane proteolipid *PMP1* (CGD identifier: C6_01530C). Both alleles of *PMP1* were deleted in the BWP17 background by using primers *PMP1*-5DR and *PMP1*-3DR to amplify (i) plasmid pDDB57 (from A. Mitchell, Carnegie Mellon University) and (ii) the plasmid pRS-ARG4ΔSpeI (from A. Mitchell, Carnegie Mellon University). First, *C. albicans* BWP17 was transformed with the *PMP1Δ*::*dpl200-URA3-dpl200* PCR amplicon using the lithium acetate method to create a *PMP1*Δ/+ strain. Then, the *PMP1Δ*::*ARG4* PCR amplicon was used to transform the *PMP1*Δ/+ strain using selective media without arginine. The transformants were screened for homologous reintegration via PCR with primers *PMP1*-5Det and *PMP1*-3Det.

### Construction of *pma1* Truncation Strains

The first allele of *PMA1* was deleted in the BWP17 background by using primers *PMA1*-5DR and *PMA1*-3DR to amplify the *dpl200-URA3-dpl200*-containing plasmid pDDB57. *C. albicans* BWP17 was transformed with the *PMA1*Δ*::dpl200-URA3-dpl200* PCR amplicon to create a *PMA1*Δ/+ strain. Then, the second allele of *PMA1* was truncated by 18, 30, 32, 34, or 38 amino acids via PCR-based gene disruption. The 5′ primers were designed with homology to the C-terminal region of *PMA1* immediately upstream of the sequence to be deleted, followed by a premature stop codon (Δ878p-5DR, Δ866p-5DR, Δ864p-5DR, Δ862p-5DR or Δ858p-5DR, see Supplemental Table 2). These primers were used in conjunction with a 3′ primer with homology to a region downstream of the *PMA1* open reading frame, Δ878p-3DR (Supplemental Table 2), to amplify the replacement cassette from the plasmid pRS-ARG4ΔSpeI (from A. Mitchell, Carnegie Mellon University). The resultant PCR amplicons were used to transform the *PMA1*Δ/+ strain using selective media without arginine. Transformants were screened for homologous reintegration via PCR with primers PMA1-qRT-5 and PMA1-3Det. In order to control for *URA3* positioning effects, the *pma1*Δ/+ strain and the *pma1Δ/Δ* truncation strains were plated to FOA agar media and the resultant FOA-resistant colonies were screened via PCR for the *PMA1/PMA1*Δ::*dpl200* genotype using primers *PMA1*-5Det and *PMA1*-3Det. Prototrophy was restored by integrating either the *URA3::HIS::ARG4*-containing plasmid CIp30 (for the *pma1*Δ/+ strain) or the *URA3::HIS*-containing plasmid CIp20 (for the *pma1Δ/Δ* truncation strains) at the *RP10* locus as previously described (Dennison et al., 2005). Because of the well-known possibility of chromosomal abnormalities with use of 5-FOA, the *URA3* marker was re-introduced into the same neutral genetic locus in all strains. Analysis of at least two strains was undertaken to ensure that phenotypic differences were consistent. The truncated region of *PMA1* was PCR amplified using primers PMA1-AMP-3 and PMA1-AMP-3, and the location of the premature stop codon was verified by Sanger sequencing of the C-terminal region of the truncated *PMA1* alleles (Eton Bioscience, San Diego, CA, United States) using primers PMA1-SEQ-5 and PMA1-SEQ-3.

### Construction of GFP- and pHLuorin-Tagged Strains

All GFP- and pHLuorin-tagged strains were generated by transformation with the positive selection marker nourseothricin. Pma1p was C-terminally tagged with green fluorescent protein (GFP) in the SC5314 background to generate Pma1p-GFP (Supplemental Table 1). First, primers PMA1-GFP-5DR and PMA1-GFP-3DR (Supplemental Table 2) were used to amplify the GFP-*NAT1* cassette from plasmid pGFP-NAT1 (from S. Bates, University of Exeter) (Milne et al., 2011); SC5314 was transformed with the resulting PCR amplicon as previously described (Milne et al., 2011) and transformants were selected for on Sabouraud-Dextrose agar containing 200 μg/mL nourseothricin (Gold Biotechnology, St. Louis, MO, United States). Primers PMA1-RT-5 and PMA1-3Det (Supplemental Table 2) were used to screen for correct integration of the *PMA1*-GFP allele. Strains tagged with cytosolic pHLuorin were constructed as previously described (Liu and Köhler, 2015) by transforming the BWP17+CIp30 control strain and the *pma1Δ/Δ* truncation strains with StuI-digested pJK1252 plasmid DNA (from J. Köhler, Boston Children’s Hospital). Vacuolar pHLuorin-tagged strains were constructed as previously described (Tournu et al., 2017) by transforming the BWP17+CIp30 control strain and the *pma1Δ/Δ* truncation strains with NgoMIV-digested pDUP3_CPP_pHL2 plasmid DNA (from G. Palmer, University of Tennessee) to generate a carboxypeptidase-Y-tagged high-fluorescence *Candida*-optimized pHLuorin2 gene.

### Plasma Membrane Isolations, Western Blots, and ATPase Activity Assays

Plasma membrane proteins were extracted via sucrose density gradient centrifugation as previously described (Cabezón et al., 2009), with some modifications. Cells grown in 5 mL cultures for 4–8 h at 30°C, 250 rpm were diluted into 1L YPD and grown overnight in 1L YPD at 30°C, 250 rpm to obtain exponentially-growing cells. Cells were harvested by centrifugation at 3500 rpm for 5 min, resuspended in protoplast pretreatment buffer (10 mM Tris-HCl pH 9.0, 5 mM EDTA pH 8.0, 1% v/v β-mercaptoethanol), and grown for 30 min at 150 rpm, 30°C. After harvesting via centrifugation, cells were washed with 2% glucose to maintain Pma1p in its glucose-activated conformation and resuspended in protoplast buffer (1.2M sorbitol, 2% glucose, 10 mM Tris-HCl pH 7.5) at a concentration of 15 OD/mL. To form protoplasts, zymolyase was added to a concentration of 1.5 units per mL and cells were grown for an additional hour at 150 rpm, 30°C. Protoplasts were collected by centrifugation at 2500 rpm for 5 min, 4°C and washed in 1.2M sorbitol in YPD. Then, the pellet was resuspended in 10 mL 0.4M sucrose, 25 mM imidazole-HCl pH 7 and harvested via centrifugation at 2500 rpm for 5 min. Protoplasts were broken by vortexing with glass beads: a 1:1 mixture of protoplasts and glass beads was suspended in 15 mL 0.4M sucrose, 25 mM imidazole-HCl pH 7 and vortexed for a total of 10 min. Glass beads and unbroken cells were removed via centrifugation at 530×*g*, +4°C, 20 min. The supernatant was centrifuged for 1 h at 30,000×*g*, 4°C using a Beckman Coulter L8-70M ultracentrifuge with a Type 70 Ti rotor. From this point forward, all media were supplemented with protease inhibitors to a final concentration of 1 μg/mL pepstatin A, 2 μg/mL chymostatin, 1 mM PMSF, 5 μg/mL aprotinin and 1μg/mL leupeptin to prevent protein degradation. Membranes were resuspended in 25 mM imidazole-HCl, pH 7 and layered on top of a discontinuous sucrose gradient (three fractions of sucrose in 25 mM imidazole-HCl pH 7; from bottom to top, 79.0, 56.5, 39.4%). A Beckman-Coulter L8-70M ultracentrifuge with a SW41Ti rotor was used to spin the gradients overnight at 150,000 ×*g*, +4°C. Plasma membranes were collected from the 56.5–79% sucrose interface, resuspended in 25 mM imidazole-HCl pH 7.5, and centrifuged for 1 h at 30,000 × g, 4°C using a Beckman-Coulter L8-70M ultracentrifuge with a Type 70Ti rotor. Purified plasma membranes were resuspended in 25 mM imidazole-HCl pH 7, 50% glycerol and stored at -20°C.

For Western blots, plasma membrane protein (50 μg) was separated by SDS-PAGE and transferred to nitrocellulose overnight at 150 mA. Pma1p was detected with a 1:5000 dilution of anti-N-terminal Pma1p rabbit polyclonal antibody (gift of Amy Chang; University of Michigan); the antibody cross-reacts with the *C. albicans* protein. Pma1p levels were then visualized using a 1:20,000 dilution of an anti-rabbit secondary antibody conjugated to an infrared dye with fluorescence at 800 nm (Anti-rabbit IR 800CW, Li-cor Inc.). Imaging, background correction, and protein quantification were completed using Li-cor Odyssey FC imager and Li-cor ImageStudio software, respectively.

To quantify ATP hydrolysis, 7.5 μg purified plasma membranes were used in an enzymatic assay in which the rate of ATP hydrolysis is coupled to the oxidation of NADH, measured as a loss of A_340_ over time (Owegi et al., 2005). Thirty micromolar of the H^+^-ATPase inhibitor ebselen was used to assess Pma1p-specific activity, and 100 nM of the V-ATPase inhibitor concanamycin A was used to ensure absence of V-ATPase-specific activity in purified plasma membranes; these concentrations are sufficient to inhibit wild-type ATPase activity in isolated plasma and vacuolar membranes *in vitro*, respectively.

We also measured zymolyase sensitivity in live yeast cells. Cells from an overnight culture were resuspended in protoplast pre-treatment buffer and grown for half an hour at 30°C, 150 rpm. After harvesting cells and washing with 2% glucose, cells were incubated in protoplast buffer with 1.5 units/mL zymolyase for an hour at 30°C as described above. Fifty microliter samples were taken at 10 min intervals and mixed with 1.3% SDS in protoplast buffer. Intact yeast survive SDS treatment whereas protoplasts lyse. Percent zymolyase sensitivity was determined by dividing OD_600_ readings at each timepoint by the starting OD_600_ reading.

### Growth and Intracellular pH

pHLuorin measurements were completed in 96-well plates. Calibration buffers (pH 5.0–9.5) were prepared as described above (see strains and media). For each strain, cells were washed in 0.9% NaCl, resuspended in a minimal volume of 0.9% NaCl, and added to calibration wells. Cells were grown for 30 min with shaking in a Synergy H1 plate reader before fluorescence readings for calibration wells (excitation = 405 and 480 nm, emission = 515 nm) were taken. Graphpad Prism 6.0 software was used to extrapolate cytosolic or vacuolar pH from the ratio of fluorescence at 405 nm to fluorescence at 480 nm in calibration curve wells.

Growth and cytosolic or vacuolar pH were measured concurrently in liquid media: pHLuorin-tagged cells were diluted to OD_600_ = 0.1 in low-fluorescence CSM (CSM prepared with YNB without amino acids, riboflavin or folic acid, pH ∼6.0). For alternate carbon source assays, cells were diluted in low-fluorescence YNB ± 2% glucose, 2% glycerol, 2% ethanol, or 1mM amino acids (pH ∼6.0). Cells were grown at 30°C using a Synergy H1 microplate reader (Biotek) with double orbital shaking at fast speed and 2 mm frequency; OD_600_ and fluorescence (excitation = 405 and 480 nm, emission = 515 nm) readings were taken at 15 min intervals. Growth curves were analyzed using Graphpad Prism: first, curves were fitted using a Weibull growth model (Weibull, 1951). Then, calculated growth rates were analyzed via one- or two-way ANOVA, as appropriate, followed by multiple-comparison post-tests. For thin-section electron microscopy, cells were grown for 16 h in complete synthetic media with glucose (glucose-replete) and without glucose (glucose-starved), then spun down and resuspended in fixative solution (10 mM NaH_2_PO_4_, 6.75 mM NaOH, 35% glutaraldehyde, 18% formaldehyde). Thin-section electron microscopy was performed at the Pathology Electron Microscopy Facility, UTHSCSA (San Antonio, TX, United States).

We also tested the ability of the Pma1p truncation strains to grow on media containing cell wall stressors (Congo Red, Calcofluor White, or SDS) or antifungal drugs (fluconazole, amphotericin B, or caspofungin). Media were prepared as previously described (Bernardo and Lee, 2010). Serial dilutions of cells were spotted onto plates as described previously (Rane et al., 2013).

### Response to Glucose

Glucose response was measured using a modification of an existing medium acidification assay where pH is measured in glucose-starved cells prior to and after glucose addition (Billack et al., 2009). Cells grown overnight in 5 mL YPD at 30°C, 250 rpm were washed twice in 10 mL ice-cold water, resuspended in 10 mL ice-cold water and incubated on ice for 30 min to induce glucose starvation. Glucose-starved cells were harvested via centrifugation at 2500 rpm for 3 min and the cell pellet was resuspended in 6.25 mM KCl. Cells in KCl were added to wells of a 96-well plate along with calibration curve wells as described above. Fluorescence (excitation = 405 and 480 nm, emission = 515 nm) readings were taken at 30 s intervals for an hour in a Synergy H1 plate reader (Biotek). The plasma membrane ATPase inhibitor ebselen was added to half the wells to a final concentration of 50 μM (a concentration sufficient to inhibit glucose uptake in wild-type cells *in vivo*) and incubated for 2 min with shaking at 30°C before reading fluorescence as described above. Then, glucose was added to all wells to a final concentration of 2% and fluorescence readings were taken at 30 s intervals for 30 min. To calculate change in pH upon glucose addition (ΔpH_i_), the cytosolic pH before glucose addition was subtracted from cytosolic pH after glucose addition. Then, ΔpH_i_ in ebselen-treated wells was subtracted from ΔpH_i_ in non-ebselen treated wells to determine ebselen-sensitive ΔpH_i_, or Pma1p-specific glucose response.

### Vacuolar Staining

To assess vacuolar morphology, vacuoles were co-stained with the lipophilic membrane dye FM4-64 and the vacuolar lumen dye CDCFDA. First, cells from an overnight culture were inoculated into 5 mL fresh unbuffered YPD (pH ∼6.8) and grown for 4 h at 30°C, 250 rpm to mid-log phase. Cells were pelleted via centrifugation and grown in 0.5 mL YPD containing 4 μM FM4-64 for 15 min at 30°C, 250 rpm. Media was changed for fresh YPD and cells were grown for another 45 min at 30°C, 250 rpm. Cells were transferred into 0.5 mL sodium citrate buffer (50 mM sodium citrate, 2% glucose, pH 5.0) containing 20μM CDCFDA and incubated at room temperature for 30 min. Stained cells were imaged with a Zeiss AxioImager M2 fluorescent microscope using DIC, Texas Red (FM4-64) and YFP (CDCFDA) filters. The number of vacuoles per cell was quantified using the cell counter feature included in FIJI software (Schindelin et al., 2012). Cells were categorized as containing one, two, three, or four or more vacuoles. Statistical analysis was conducted in Graphpad Prism 6.0 software using a chi-square goodness-of-fit test to compare expected versus observed distributions, with expected distributions equaling those observed in the BWP17+CIp30 control.

We tested the ability of Pma1p truncation mutants to grow in the presence of the V-ATPase inhibitor bafilomycin A1 at a sub-lethal concentration. Wild-type and Pma1 truncation mutant cells were diluted to OD_600_ = 0.1 in CSM with and without 100 μM bafilomycin A1. Cells were grown at 30°C using a Synergy H1 microplate reader (Biotek) with double orbital shaking at fast speed and 2 mm frequency; OD_600_ readings were taken at 15 min intervals.

### Filamentation, Secretion, and Biofilm Formation Assays

Filamentation was assessed in liquid media and on solid media. Filamentation in liquid media was tested in RPMI/L-glutamine/MOPS pH 7.0. Media was inoculated with cells from overnight cultures to a starting density of 5 × 10^6^ cells per mL, and cells were grown at 37°C, 200 rpm for 2–24 h. Cells were visualized via DIC microscopy using a Zeiss EC Plan-NeoFluar 10X objective (Carl Zeiss AG, Germany) at selected time points. Solid media tested were YPD+10% fetal calf serum (FCS); Medium 199 supplemented with L-glutamine (M199); Spider medium as previously described (Liu et al., 1994); and RPMI/L-glutamine. Spider media was prepared with 1.35% (w/v) agar. Three microliter cells from overnight cultures were OD_600_-matched before spotting to agar plates and incubating at 37°C for 5 days.

To examine the distribution of Pma1p in filaments, Pma1-GFP cells from an overnight culture were washed three times in 1XPBS and diluted to a concentration of 5 × 10^6^ cells per mL in YNB, 2% glucose + 20% FCS. Cells were grown for 4 h at 37°C, 200 rpm, with samples taken for microscopy at 0, 0.5, 1, 2, and 4 h timepoints. Cells were imaged on an Olympus confocal microscope; maximum intensity Z-stacks comprised of 0.2-micron slices are presented to show that the observed non-homogenous distribution of Pma1-GFP in filaments was not due to hyphal cells extending out of the microscope’s field of view.

Aspartyl protease and phospholipase secretion was assessed on solid media. Extracellular protease secretion was assayed on unbuffered bovine serum albumin (BSA) plates (Crandall and Edwards, 1987), and phospholipase secretion was assayed on egg yolk agar plates (Fu et al., 1997). Three microliter OD_600_-matched overnight culture cells were spotted onto plates. BSA plates were incubated at 30°C for 48 h, and egg yolk agar plates were incubated at 37°C for 120 h.

Biofilm formation was tested in a static 96-well microplate model as previously described (Ramage and López-Ribot, 2005). Biofilm mass was assessed via crystal violet staining as previously described (Jin et al., 2003). The XTT reduction assay was also used to assess biofilm metabolic activity (Ramage and López-Ribot, 2005).

### Calcofluor White Staining

Cell wall chitin was stained with Calcofluor white as previously described (Pringle, 1991). Cells from an overnight culture were diluted 1:1000 into 5 mL fresh YPD+uridine and grown for 16 h at 30°C, 250 rpm to late log phase. Cells were fixed by adding formaldehyde to a final concentration of 3.7% and incubating for an additional 45 min at 30°C, 250 rpm. Cells were washed twice with 1mL water before resuspension in 0.5 mL Calcofluor white stain (1 mg/mL Calcofluor white). Cells were stained at room temperature for 5 min and washed twice with water before imaging on an Olympus confocal microscope using a 405 nm laser. Images taken were Z-stacks comprised of 0.2 micron-thick slices. Bud scars were scored as axial, bipolar or random as previously described (Ni and Snyder, 2001). Only cells with three or more bud scars were scored. Cells with bud scars clustered on one side of the cell were scored as axial, cells with bud scars on opposite sides of the cell were scored as bipolar and cells with bud scars elsewhere were scored as random. Statistical analysis was conducted in Graphpad Prism 8.0 software using a chi-square goodness-of-fit test to compare expected versus observed distributions, with expected distributions equaling those observed in the BWP17+CIp30 control.

### Statistical Analyses

Where required, results were analyzed for statistical differences using GraphPad Prism software. Results were considered significant if *p* < 0.05 compared to all other treatments.

## Results

### Genetic Alteration of *PMA1*

We initially tried several strategies to genetically analyze *PMA1*. First, we constructed a tetR-*PMA1* strain in which one allele of *PMA1* was deleted and the second allele placed under the control of a doxycycline-repressible promoter. This strain proved difficult to study for two reasons. RT-PCR revealed that *PMA1* expression was increased in the tetR-*PMA1* strain in the absence of doxycycline due to the constitutive nature of the *tet* promoter (Supplemental Figure 1). Overexpression of *PMA1* in tetR-*PMA1* led to several unexpected phenotypes, including decreased growth compared to wild-type at low pH or in unbuffered media (CSM pH 4, pH 5 and unbuffered CSM) and increased growth at alkaline pH (CSM pH 7.5 or 8.5; data not shown). The tetR-*PMA1* strain also exhibited impaired filamentation in the absence of doxycycline on weak-inducing media (Spider, RPMI, and M199 agar; Supplemental Figure 1). Secondly, in the presence of doxycycline the tetR-*PMA1* strain failed to grow in low pH media or unbuffered media and grew poorly in alkaline pH media (Supplemental Table 3). However, cell viability assays revealed that the tetR-*PMA1* strain retained close to 100% viability even after 24 h of treatment with doxycycline (Supplemental Figure 1). In *S. cerevisiae*, Pma1p inheritance during budding is asymmetric, such that daughter cells inherit little Pma1p from mother cells; Pma1p levels remain low until daughter cells are able to independently synthesize Pma1p (Henderson et al., 2014). In addition, turnover of Pma1p is slow, with a half-life close to 12 h (Benito et al., 1991). We suspect these phenomena account for the fact that viability is retained in the absence of growth in the tetR-*PMA1* strain in the presence of doxycycline. Mother cells may survive by retaining previously-synthesized Pma1p for up to 24 h after *PMA1* expression is repressed, while daughter cells are inviable due to the inability to produce new Pma1p protein (Dechant et al., 2014).

Previous work in *S. cerevisiae* identified two small proteolipids that associate with the plasma membrane H^+^-ATPase, Pmp1p, and Pmp2p (plasma membrane proteolipid) (Navarre et al., 1992, 1994). *PMP1* and *PMP2* are orthologs that arose from the whole genome duplication. Deletion of both *PMP1* and *PMP2* decreases the ATPase-specific activity of the plasma membrane H^+^-ATPase by 50% without altering the amount of Pma1p at the plasma membrane (Navarre et al., 1994). A BLAST search of the *Candida* Genome Database using either *PMP1* or *PMP2* as a query revealed a single gene, *orf19.3432.1* (CGD identifier: C6_01530C) (Skrzypek et al., 2017). We constructed a *C. albicans pmp1*Δ/Δ null mutant strain by deleting both alleles of *orf19.3432.1* from the *C. albicans* genome. *C. albicans pmp1*Δ/Δ unexpectedly had no significant differences in cell growth at varying pH, filamentation or biofilm formation compared to wild-type (data not shown).

Another method to disrupt Pma1p is C-terminal truncation of the enzyme (Portillo and Serrano, 1988; Mason et al., 2014). We generated a series of Pma1 truncation mutants in which the enzyme was truncated at the C-terminus by 18–38 amino acids. These truncation mutant strains are, from least to most truncated, Δ878 (truncated by 18 amino acids), Δ866 (truncated by 30 amino acids), Δ864 (truncated by 32 amino acids), Δ862 (truncated by 34 amino acids), and Δ858 (truncated by 38 amino acids). For a full list of strains used in this study, see Supplemental Table 1.

### Assessment of Pma1p Levels and ATPase-Specific Activity in Purified Plasma Membranes

**FIGURE 1:**
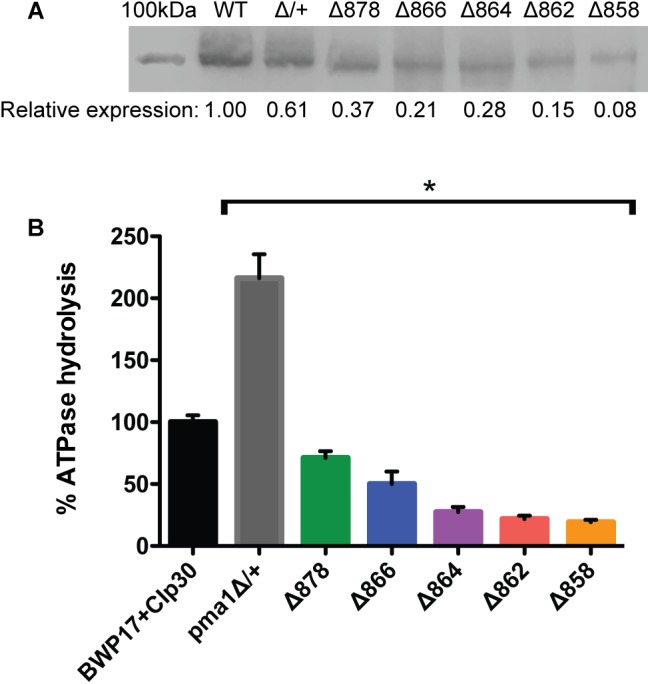
Pma1p truncation mutants have decreased plasma membrane Pma1p levels and decreased plasma membrane ATPase activity. Plasma membranes were isolated from exponentially growing cells via zymolyase- and glass bead-mediated cell wall disruption followed by density-gradient sucrose centrifugation. Plasma membranes were isolated independently in quadruplicate. **(A)** Western blot using a rabbit primary antibody raised against the N-terminus of *S. cerevisiae* Pma1p and a goat anti-rabbit secondary antibody conjugated to an IR dye with fluorescence at 800 nm. 100 kDa indicates a 100 kDa marker band (Precision Plus Protein ladder, Bio-Rad) stained with Coomassie blue and captured via measuring fluorescence at 700 nm. The expected size of *C. albicans* Pma1p is approximately 100 kDa. WT indicates the wild-type control strain, BWP17+CIp30. Western blots were performed independently in triplicate. Relative expression levels were quantified using Licor ImageStudio software and normalized to expression in the wild-type control strain. Pma1p levels decrease with increasing Pma1p truncation in isolated plasma membranes. Pma1p levels are also decreased in a strain heterozygous for *PMA1*. **(B)** Percent ATPase-specific activity in isolated plasma membranes as compared to ATPase-specific activity in the BWP17+CIp30 wild-type control. ATPase hydrolysis activity was measured using an enzymatic assay in which the rate of ATP hydrolysis is coupled to the oxidation of NADH, measured as a loss of A_340_ over time. ATPase assays were performed four times independently, with four technical replicates each time. The average BWP17+CIp30 ATPase-specific activity was 0.30 μmol Pi/min/mg. Increasing C-terminal truncation of Pma1p corresponds to decreased ATPase-specific activity in purified plasma membranes. Deletion of a single allele of *PMA1* results in a compensatory increase in ATPase-specific activity. Asterisk (^∗^) denotes statistical significance (*p* > 0.01) compared to the BWP17+CIp30 wild-type control in a one-way ANOVA.

We isolated plasma membrane protein from exponentially-growing wild-type and Pma1p truncation mutant cells via density-gradient centrifugation and measured (i) Pma1p levels via Western blotting with an antibody raised against the N-terminus of the Pma1p protein, and (ii) ATPase-specific activity spectrophotometrically using a coupled enzymatic assay (Owegi et al., 2005). Increasing Pma1p truncation corresponded to decreased Pma1p levels and decreased ATPase-specific activity in purified plasma membranes (Figure 1). Truncation of Pma1p by only 18 amino acids in the Δ878 strain decreased Pma1p amounts and ATPase-specific activity in isolated plasma membranes by 63 and 44.1%, respectively. Truncation by 38 amino acids in the Δ858 strain led to decreases in Pma1p levels and ATPase-specific activity in plasma membranes of more than 90 and 75%, respectively. Interestingly, ATPase-specific activity levels were higher than relative Pma1p levels in plasma membranes isolated from all five Pma1p truncation mutants. We also measured Pma1p levels and ATPase-specific activity in plasma membranes isolated from a strain heterozygous for *PMA1, pma1*Δ/+ (Figure 1). Although the amount of Pma1 at the plasma membrane decreased by 39% in *pma1*Δ/+ cells, ATPase-specific activity was significantly increased compared to wild type. The increase was almost exactly twofold (216%). We further characterized the Pma1 heterozygote in several phenotypic assays, including growth, stress response, and filamentation (data not shown); the Pma1 heterozygote showed no phenotypic difference from wild-type under any condition tested. Together these results indicate that cells can increase the activity of the Pma1p enzyme to help compensate for decreases in the amount of Pma1p at the plasma membrane.

While preparing purified plasma membranes, we noticed a difference in the sensitivity of Pma1p truncation mutant strains to the cell wall disrupting enzyme zymolyase. Zymolyase contains a mixture of β-1,3-glucanase and mannanase. Truncation of Pma1p by 18–34 amino acids increased resistance to zymolyase treatment. After 1 h, 66.6% of wild-type cells treated with zymolyase formed protoplasts. In contrast, in the Δ878 strain (lacking 18 aa), only 12.5% of cells formed protoplasts. The level of zymolyase resistance decreased with further Pma1p truncation, such that in the Δ862 strain (truncated by 34 amino acids) 33.8% of cells were lysed after 1 h. In the Δ858 strain (lacking 38 aa), zymolyase sensitivity was closer to wild-type levels, with 70.7% of cells lysed after 1 h incubation with zymolyase. These results may indicate differences in cell wall composition in Pma1p truncation mutants; further study is warranted.

### Analyses of Growth and Cytosolic pH in Pma1p Truncation Mutants

**FIGURE 2:**
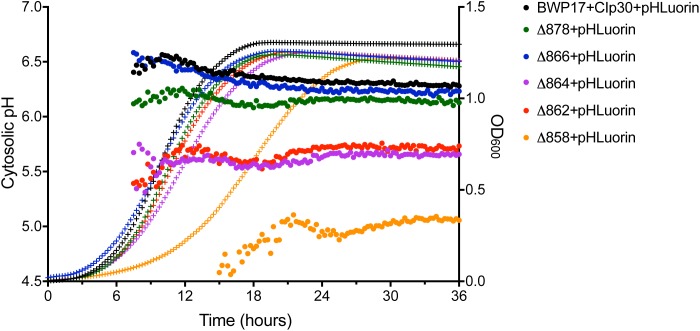
Growth and cytosolic pH in Pma1p truncation mutants. Growth and cytosolic pH were measured concurrently in Pma1p truncation mutants by growing cells in YNB with 2% glucose in a plate reader with OD_600_ readings and cytosolic pH readings taken at 15 min intervals. Cytosolic pH is plotted on the left *y*-axis and is represented by closed circles. OD_600_ is plotted on the right *y*-axis and is represented by + symbols. Increased Pma1 truncation is correlated with decreased growth and increased cytosolic acidification. Graph represents the average of four individual experiments.

Cytosolic pH varies with growth phase in *C. albicans*; cytosolic pH is higher in exponentially-growing cells compared to stationary-phase cells (Tournu et al., 2017). We measured cytosolic pH alongside growth in Pma1p truncation mutants in complete synthetic media with 2% glucose to determine whether growth-phase-specific cytosolic pH is affected by decreased ATPase-specific activity. In accordance with previous studies, we saw a decrease in cytosolic pH upon transition to stationary phase in wild-type cells. Increasing Pma1p truncation correlates with decreased growth rates and more acidic cytosols (Figure 2). The growth defect in the Δ858 strain (lacking 38 aa) is particularly dramatic, whereas the growth of the Δ878 strain (lacking 18 aa) approached wild-type levels (Figure 2) despite a > 40% loss of plasma membrane ATPase-specific activity (Figure 1). The degree of cytosolic acidification we observed was remarkable. In the Δ864, Δ862, and Δ858 mutants (lacking 32, 34, and 38 aa, respectively), the pH of the cytosol remained close to pH 5.5 (Δ864 and Δ862) or pH 5.0 (Δ858) for the duration of the experiment regardless of growth phase. In wild-type cells, cytosolic pH increases during exponential growth to pH ∼6.5 and decreases as cultures reach stationary phase, to pH ∼6.25. Similarly, in strains with truncations of Pma1p by 18–32 amino acids, cytosolic pH decreases upon transition to stationary phase. Truncation of Pma1p by 34 amino acids or more disrupts this growth stage-dependent phenotype; cytosolic pH fails to decrease upon transition to stationary phase in Pma1p truncation mutants Δ864, Δ862 and Δ858 compared to exponentially growing cells (Figure 2).

In *S. cerevisiae*, growth in acidic media leads to a two- to threefold increase in plasma membrane H^+^-ATPase activity (Eraso and Gancedo, 1987). Further, we previously noted a decrease in growth at acidic pH upon overexpression of *PMA1* in the tetR-*PMA1* strain. We measured growth of the Pma1p truncation mutants at varying environmental pH, and in media containing alternate carbon sources. For varying pH assays, CSM media was buffered to pH 4.0 – pH 8.5. For alternate carbon source assays, YNB media was prepared with 2% ethanol, 2% glycerol, or an amino acid as the sole carbon source. Amino acid media tested were YNB ± 1mM alanine, arginine, cysteine, glutamine, histidine, leucine, lysine, methionine, phenylalanine, proline, or threonine. The delay in growth upon Pma1p truncation observed in defined media with glucose was neither increased nor ameliorated under these conditions (data not shown), indicating a global role for *PMA1* in growth independent of carbon source or external pH level. Pma1p truncation mutants also grew as well on media containing sub-inhibitory concentrations of cell wall stressors (Congo Red, Calcofluor White, or SDS) or antifungal drugs (fluconazole, amphotericin B, or caspofungin) as on complete synthetic medium compared to wild-type (data not shown).

**FIGURE 3:**
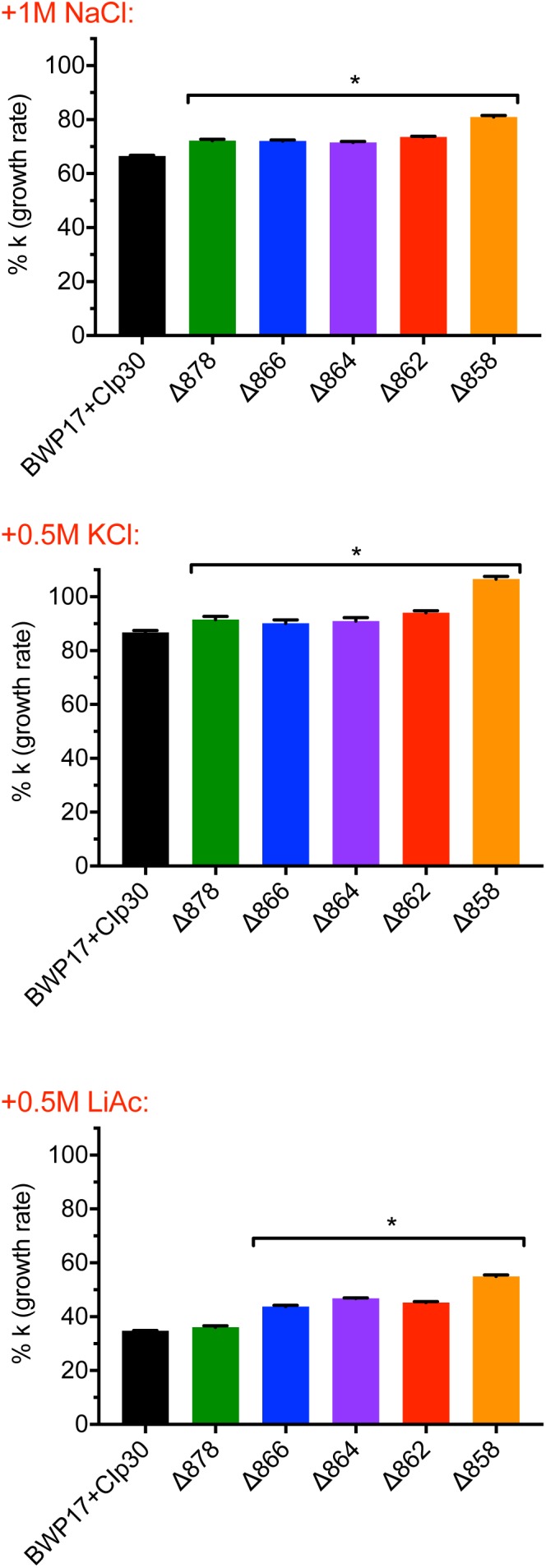
Growth of Pma1p truncation mutants in the presence of cations. Growth was measured in Pma1p truncation mutants by growing cells in complete synthetic media with 2% glucose ± 1M NaCl, 0.5M KCl or 0.5M LiOAc (lithium acetate) in a plate reader with optical density at 600 nm (OD_600_) readings taken at 15 min intervals. Growth rate (k) was calculated using Graphpad Prism software, and %k was calculated for each strain by comparing growth in the presence of salts to growth in media without salts added. Increasing Pma1p truncation decreases the effect of cation addition on growth. Each graph represents a single representative experiment; experiments were completed twice. Growth rates were analyzed for statistical differences compared to growth of the BWP17+CIp30 control in the presence of each salt via two-way ANOVA followed by a multiple comparison post-test. Asterisks denote statistical significance (*p* > 0.01).

The pH gradient generated by Pma1p at the plasma membrane is used by cation-proton antiporters to maintain ion balance. Therefore, we measured growth and cytosolic pH in Pma1p truncation mutants in the presence of high cation (Na^+^, K^+^, Li^+^) concentrations. High cation concentrations significantly decreased growth. Increasing Pma1p truncation mitigated the effect of cation addition on growth (Figure 3). Interestingly, the cytosol of wild-type cells treated with cations was markedly more acidic compared to untreated wild-type cells upon reaching stationary phase; cytosolic pH dropped by 0.115 ± 0.043 units in stationary-phase cells treated with 1M NaCl compared to stationary-phase cells without cation treatment, by 0.465 ± 0.030 units in cells treated with 1M KCl, and by 0.092 ± 0.024 units in cells treated with 0.25M lithium acetate. This acidification is likely due to more rapid inhibition of wild-type Pma1p compared to truncated Pma1p, as wild-type Pma1p pump activity establishes a positive external membrane potential, more so than truncated Pma1p. Consequently, addition of external cations will likely inhibit wild-type Pma1p activity more quickly, due to a higher baseline membrane potential. Alternatively, a component of this acidification may be caused by activation of vacuolar cation/proton antiporters under high salt conditions. Such transporters, e.g., Nhx1p, Vcx1p, or Vnx1p, function predominantly at the vacuole. Cation sequestration in the vacuole requires the exchange of vacuolar protons for cytoplasmic cations; such activity would be expected to decrease the pH of the cytosol. The drop in cytosolic pH upon transition to stationary phase dissipated with increasing Pma1p truncation (data not shown), perhaps due to the already-acidified cytosol present upon Pma1p truncation (Figure 2). These data may indicate that the decrease in growth rate upon treatment with high concentrations of cations is due at least in part to cytosolic acidification.

### Pma1p Is Regulated by Glucose Availability

**FIGURE 4:**
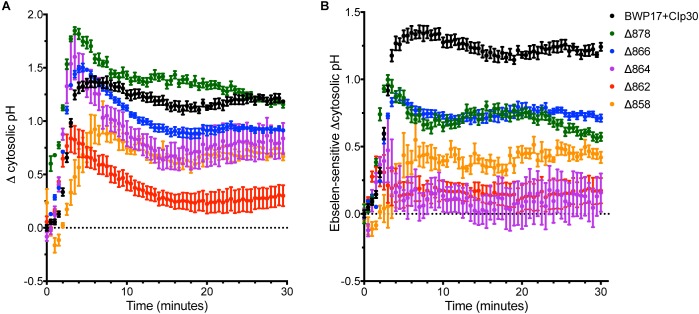
Cytosolic pH alkalinization in response to glucose addition is altered in Pma1p truncation mutants. After a 30 min period of glucose starvation, glucose was added to wild-type and Pma1p truncation mutant cells and cytosolic pH was measured at 30 s intervals. The addition of glucose to glucose-starved cells causes a rapid and dramatic increase in cytosolic pH of nearly 1.5 units in wild-type cells. Increasing Pma1p truncation decreases this glucose-induced pH change. Graphs represent a single representative experiment; experiments were conducted at least four times. **(A)** Change in pH upon glucose addition (ΔpH_i_) was calculated by subtracting cytosolic pH immediately prior to glucose addition from cytosolic pH at each timepoint after glucose addition. The Δ862 and Δ858 strains exhibit lower cytosolic pH response to glucose than the wild-type control strain. **(B)** Ebselen-sensitive ΔpH_i_, or Pma1p-specific glucose response, was calculated by subtracting ΔpH_i_ in wells treated with 50 μM of the H^+^-ATPase inhibitor ebselen from ΔpH_i_ in non-ebselen treated wells at each timepoint. Ebselen-sensitive ΔpH_i_ is significantly lower than ΔpH_i_, potentially indicating the presence of compensatory cytosolic alkalinization mechanisms in Pma1p truncation mutants.

Glucose alters the kinetics of the Pma1p enzyme; in *S. cerevisiae*, the glucose-activated conformation of the ATPase has 10-fold higher activity, a more alkaline pH optimum, and better ATP affinity (Serrano, 1983). However, the kinetic response of *C. albicans* to glucose is greatly decreased compared to *S. cerevisiae* (Monk et al., 1991). Glucose also alters the amount of protein at the plasma membrane. In *C. albicans*, glucose-starved cells contain half the amount of Pma1p as growing cells (Monk et al., 1993). The addition of glucose to starved *C. albicans* cells rapidly activates the ATPase, as indicated by a 10-fold increase in ATPase-specific activity followed by a sharp increase in cytosolic pH (Monk et al., 1993). Further, C-terminal truncation of Pma1p by 11 amino acids in *S. cerevisiae* removes the glucose regulatory domain and leads to constitutive activation of the enzyme (Portillo et al., 1989). Therefore, we tested the ability of Pma1 truncation mutants to respond to glucose addition (Figure 4). The Δ862 and Δ858 strains exhibited decreased cytosolic pH response to glucose compared to the wild-type control strain. The Δ878 strain, in which 18 amino acids have been deleted from the C-terminus, had a more rapid response to glucose than wild-type cells. To determine whether other mechanisms besides Pma1p activity were involved in the response to glucose in the Pma1p truncation mutants, we treated cells with the H^+^-ATPase inhibitor ebselen prior to glucose addition (Figure 4B). The proportion of the pH response due specifically to Pma1p activity was calculated by subtracting ΔpH_i_ in wells treated with the H^+^-ATPase inhibitor ebselen from ΔpH_i_ in non-ebselen treated wells at each timepoint. Ebselen-sensitive ΔpH_i_ was significantly lower than ΔpH_i_ upon glucose addition in Pma1p truncation mutants but not wild-type cells. These findings may indicate the presence of compensatory cytosolic alkalinization mechanisms in Pma1p truncation mutants.

**FIGURE 5:**
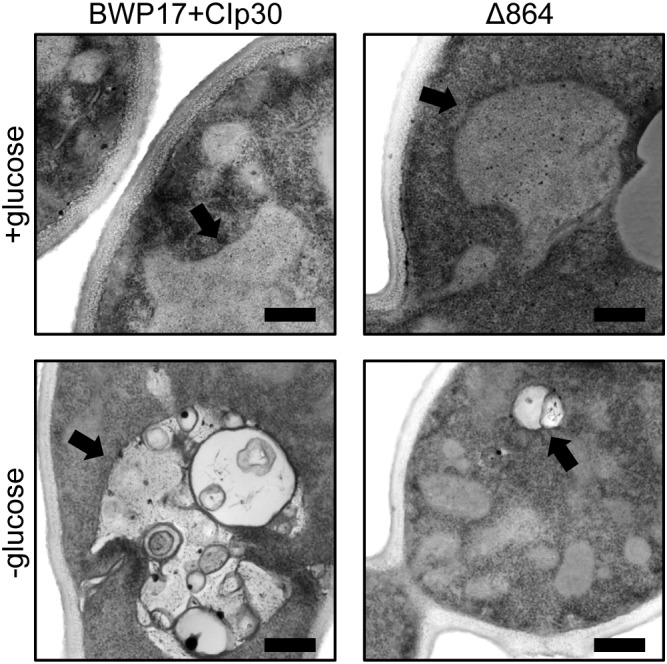
Thin-section electron microscopy reveals differences in cell wall structure and vacuolar morphology in a Pma1p truncation mutant. Wild-type and Δ864 cells were grown for 16 h under glucose-replete and glucose-starved conditions, then examined via thin-section electron microscopy. In each panel, the black arrow is pointing to the vacuole. Scale bar indicates 400 nm.

We further examined the effects of glucose availability in a Pma1p truncation mutant using thin-section electron microscopy (Figure 5). Wild-type and Δ864 cells were grown under glucose-replete and glucose-starved conditions, then examined via thin-section electron microscopy. In the presence of glucose, the cell wall of the Δ864 strain was less dense and less organized than that of the wild-type control. No differences in vacuolar morphology were evident. Upon glucose starvation, the cell wall of both wild-type and Δ864 cells appeared less dense than under glucose-replete conditions. This could suggest that Pma1p truncation mimics glucose depletion, even when glucose is plentiful. Further, in wild-type cells, vacuoles were enlarged and appear to be filled with organellar material (most likely autophagic bodies). In Δ864 cells, vacuoles were scarce (data not shown). When present, vacuoles were much smaller than wild-type vacuoles, and fewer autophagic bodies were observable (Figure 5). The defect in vacuolar morphology points to dysregulation of glucose metabolism and possibly autophagy upon Pma1p truncation. The observed differences in cell wall structure in the Δ864 strain, combined with the differences in zymolyase sensitivity we observed upon Pma1p truncation, imply that upon Pma1p truncation, overall cell wall density and organization decreases but is offset by increases in the amounts of β-1,3-glucan and/or mannan, the targets of the zymolyase enzyme.

### Pma1p Truncation Affects Vacuolar pH and Vacuolar Morphology

We measured vacuolar pH in exponentially-growing cells in glucose-replete conditions using a pHLuorin derivative that is targeted to the vacuole (Tournu et al., 2017). Under these conditions, vacuolar pH was significantly decreased in all five Pma1p truncation mutant strains as compared to the BWP17+CIp30 wild-type control (data not shown). This phenotype is likely caused by a net increase in intracellular protons when Pma1p activity is lowered. However, because the pHLuorin probe loses sensitivity below pH ∼ 5.0 (Miesenböck et al., 1998), we were unable to quantify the degree of vacuolar acidification upon Pma1p truncation. BCECF, a fluorophore commonly used for vacuolar pH measurements (Diakov et al., 2013), has similar limitations.

**FIGURE 6:**
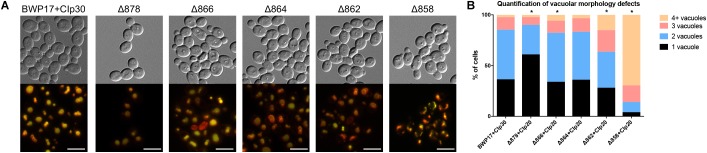
Pma1p is involved in vacuolar morphology. **(A)** Vacuoles were visualized by co-staining with the vacuolar dyes FM4-64 (membrane, pseudo-colored red) and CDCFDA (lumen, pseudo-colored yellow). Differences in vacuole appearance are most dramatic in the Δ858 strain, which contains numerous small vacuoles as compared to the BWP17+CIp30 wild-type control. Scale bars indicate 50 μm. **(B)** Quantification of vacuole number in FM4-64 and CDCFDA-stained cells. Cells were categorized as containing one, two, three, or four or more vacuoles. Statistical significance was determined using a chi-square goodness-of-fit test comparing vacuole number distribution in the Pma1p truncation mutant strains to vacuole number distribution in the BWP17+CIp30 wild-type control strain. Vacuole number distribution is significantly shifted in the Δ878, Δ866, Δ862, and Δ858 strains, with the Δ878 strain exhibiting a shift toward cells containing only one vacuole and the latter strains exhibiting a shift toward cells containing three or more vacuoles. Graph represents a single representative experiment; experiment was conducted twice.

Wild-type cells grown in unbuffered YPD media to mid-log phase contained one to two large vacuoles (Figure 6). Truncation of Pma1p by 18 amino acids led to an increase in the number of cells containing a single, enlarged vacuole. Conversely, truncation of Pma1p by 34 amino acids or more led to an increase in the number of cells containing three or more vacuoles. Truncation of Pma1p by 38 amino acids had the most dramatic effect, with most cells containing four or more small vacuoles. Vacuolar fission is the fragmentation of one vacuole into smaller daughter vacuoles. Vacuolar fusion is a related process in which daughter vacuoles fuse membranes to form a larger organelle. Coordinated cycling of these two processes occurs throughout the cell cycle and in response to changing environmental conditions. We observed a potential vacuolar fission defect in the Δ878 strain and a potential fusion defect in the Δ862 and Δ858 strains. The distribution of vacuole number was also significantly altered in the Δ866 strain, with a slight increase in the number of cells with three or more vacuoles. Interestingly, comparison of vacuolar morphology data with measurements of vacuolar pH revealed that strains with potential vacuolar fusion defects had more acidic vacuoles (data not shown). These findings are in line with our previous studies of V-ATPase genes in *C. albicans* (Rane et al., 2013, 2014), where *vma* mutants have alkalinized vacuoles and vacuolar fission defects. The observed inverse phenotype, where acidic vacuoles are correlated with vacuolar fission defects, indicates that vacuolar pH may be a direct regulator of vacuolar fission and fusion.

V-ATPase mutants have acidified cytosols due to altered trafficking of Pma1p (Tarsio et al., 2011; Smardon and Kane, 2014). We tested the growth of Pma1p truncation mutants in the presence of inhibitory concentrations of the V-ATPase-specific inhibitor bafilomycin A1 (Table 1). Pma1p truncation attenuated the effects of bafilomycin A1 on growth. Under normal conditions, bafilomycin affects cytosolic pH and growth (Table 1; Bowman et al., 1988), presumably in part through effects on Pma1p. The growth rate of control strain BWP17+CIp30 was significantly decreased in the presence of bafilomycin. A statistical difference in growth rate was also observed in all of the Pma1p truncation strains except the Δ858 strain (truncated by 38 aa) in the presence of bafilomycin, however, the degree of difference between growth rates decreased with increasing Pma1p truncation. These results indicate that the anti-growth activity of bafilomycin may be partially caused by its effects on cytosolic rather than vacuolar pH. We hypothesize that the reduction of cytosolic pH is already so extreme in Pma1p truncation mutants that bafilomycin addition has little additional effect. Another possibility is that the low cytosolic pH in Pma1p truncation mutants leads to compensatory vacuolar acidification, which reduces the effect of bafilomycin treatment.

**Table 1:** Pma1p truncation masks the effects of bafilomycin A1 on growth.

	-bafilomycin	+bafilomycin
BWP17+CIp30	0.2173 ± 0.0010	0.1745 ± 0.0009^∗^
Δ878	0.2214 ± 0.0010	0.2117 ± 0.0013^∗^
Δ866	0.2170 ± 0.0009	0.1979 ± 0.0007^∗^
Δ864	0.1792 ± 0.0008	0.1675 ± 0.0009^∗^
Δ862	0.1947 ± 0.0009	0.1914 ± 0.0009^∗^
Δ858	0.1306 ± 0.0011	0.1311 ± 0.0008

*Growth rates with and without the V-ATPase specific inhibitor bafilomycin were measured in Pma1p truncation mutants by growing cells in CSM with 2% glucose in a plate reader with OD_*600*_ readings taken at 15 min intervals. Growth curves were analyzed using Graphpad Prism: first, curves were fitted using a Weibull growth model (Weibull, 1951). Then, calculated growth rates were analyzed two-way ANOVA, as appropriate, followed by multiple-comparison post-tests. Data represents the average values of three independent experiments. Addition of bafilomycin did not reduce growth in the Δ858 strain (truncated by 38 aa, respectively). ^∗^Indicate statistical significance compared to the growth rate of the same strain without bafilomycin.*

### *PMA1* Has a Role in Cell Polarity and Filamentation

We next sought to determine whether cytosolic pH homeostasis contributes to *Candida albicans* virulence. We used our set of Pma1p truncation mutants to assess the role of *PMA1* in three major virulence-related phenotypes: secretion, filamentation and biofilm formation. Truncation of Pma1p did not affect secretion of aspartyl proteases or phospholipases (data not shown). We assessed biofilm formation in Pma1p truncation mutants and found that truncation of Pma1p led to a significant decrease in biofilm mass as measured by crystal violet assay (Table 2). We also measured biofilm metabolic activity via the XTT assay (data not shown), however, we observed strain-specific differences in XTT metabolism in the Pma1p truncation mutants that obscure interpretation of this assay. Decreased biofilm formation upon Pma1p truncation is unsurprising due to the role of *PMA1* in growth (Figure 2), and no major structural defects in biofilms formed by Pma1p truncation mutants were found (data not shown). We conclude that Pma1p does not play a specific role in biofilm formation beyond a more general role in cell growth.

**Table 2:** Biofilm formation by Pma1p truncation mutants as measured by crystal violet assay.

Strain	Mean OD_550_ ± SD^a^
BWP17+CIp30	0.2927 ± 0.1152
Δ878	0.2775 ± 0.0922
Δ866	0.2638 ± 0.0587
Δ864	0.2638 ± 0.0587
Δ862	0.3657 ± 0.0674
Δ858	0.2214 ± 0.0929

*^a^OD_550_ corresponds to optical density at 550 nm, the wavelength at which crystal violet absorbs. OD_550_ is a proxy measure for biofilm mass.*

**FIGURE 7:**
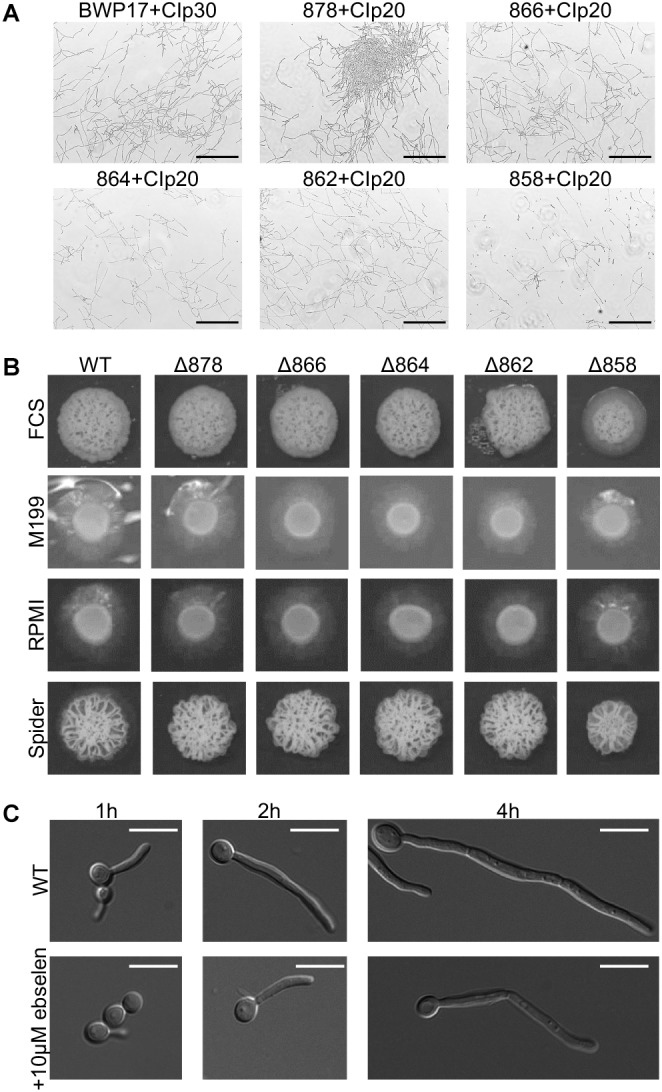
Pma1p truncation affects filamentation capacity. **(A)** Cells were grown in liquid RPMI at 37°C, 200 rpm for 24 h and imaged via light microscopy. Increasing Pma1p truncation leads to the formation of fewer and shorter filaments than the wild-type control. Scale bars indicate 200 μm. **(B)** Filamentation on hyphal-inducing agar plates. Three microliter OD-corrected cells from overnight cultures were spotted onto YPD+FCS, M199, RPMI, and Spider agar plates and incubated at 37°C for 5 days. Increasing Pma1p truncation leads to a modest defect in filamentation on FCS, M199, and RPMI agar plates. WT indicates the wild-type strain BWP17+CIp30. **(C)** Filamentation in the presence and absence of the Pma1p inhibitor ebselen at a concentration sub-inhibitory for growth. Cells were grown in liquid RPMI at 37°C for 24 h and imaged at select timepoints via light microscopy. Addition of 10 μM ebselen to the growth media decreases hyphal length. Scale bars indicate 10 μm.

**FIGURE 8:**
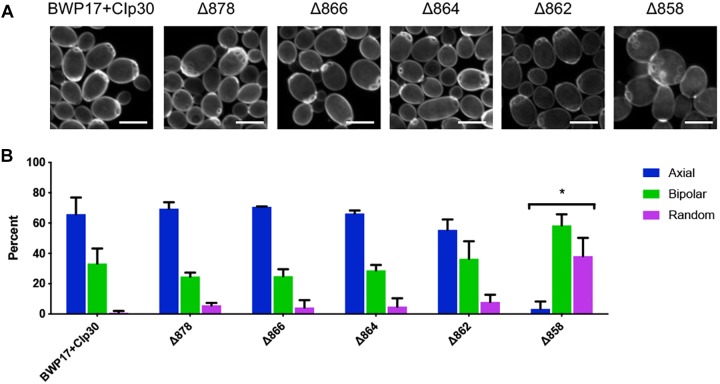
Pma1p truncation affects bud scar distribution. **(A)** Pma1p truncation mutants were stained with the chitin-binding dye Calcofluor white. Cells were imaged using a confocal microscope. A Z-stack comprised of 0.2-micron thick slices is shown. Scale bars indicate 5 μm. **(B)** Bud scar distribution was scored as axial, bipolar or random. These experiments were completed twice; both replicate experiments are represented here. Pma1p truncation significantly increases bipolar or random budding. Strains were analyzed for statistical differences compared to budding patterns of the BWP17+CIp30 control via two-way ANOVA followed by a multiple comparison post-test. Asterisks denote statistical significance (*p* > 0.01).

**FIGURE 9:**
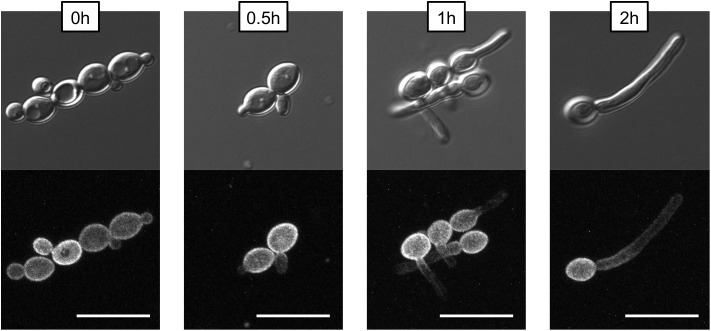
Non-homogenous distribution of Pma1p in hyphae. A wild-type strain in which *PMA1* was C-terminally tagged with GFP was diluted to a concentration of 5 × 10^6^ cells per mL and grown in YNB+20% FCS for 4 h. Cells were imaged using a confocal microscope to demonstrate that hyphal tips are not simply out of the field of view. For the GFP fluorescent image, a Z-stack comprised of 0.2-micron thick slices is shown. Pma1-GFP is distributed evenly across the plasma membrane in yeast cells, but is less prominent along the hyphae of filamenting cells. Scale bars indicate 10 μm.

One key *C. albicans* virulence trait is the ability to switch between hyphal and yeast forms. Cytosolic pH and filamentation are thought to be linked (Stewart et al., 1988); therefore we tested filamentation capacity in Pma1p truncation mutants. Increasing Pma1p truncation led to decreased hyphal length under some but not all conditions (Figure 7A,B). Further, we chemically inhibited Pma1p during hyphal-induction with ebselen, at a concentration sub-inhibitory for growth (Chan et al., 2007). Growth in the presence of 10 μM ebselen led to decreased hyphal length (Figure 7C). Hyphal formation is a form of polarized growth that depends on intact cell polarity machinery. We used the chitin stain Calcofluor white to examine bud scar distribution as a proxy measure for the establishment of cell polarity in Pma1p truncation mutant cells (Figure 8). In wild-type cells, the site of budding occurs along the cell’s poles. Bud scars predominantly present as axial (clustered together in a ring around one of the cell’s poles) or bipolar (clustered in rings along both cell poles, on opposite sides of the cell). Pma1p truncation significantly increased the amount of bipolar or randomly budded cells (Figure 8), indicating a disruption in the functionality of the cell polarity machinery. These differences were only statistically significant in the Δ858 strain (truncated by 38 aa). Lastly, we constructed a strain in which Pma1p was GFP-tagged at the C-terminus in *C. albicans* and examined Pma1p localization in emerging hyphae (Figure 9). Pma1-GFP is most highly concentrated at the plasma membrane of the yeast cell. Pma1-GFP decreases in concentration along the length of the growing hyphae, with the lowest amount of Pma1-GFP visible at the hyphal tip. These results suggest that Pma1p remains concentrated in stable microdomains in the mother cell, perhaps analogous to Pma1p distribution seen in mother cells and buds in *S. cerevisiae*. Whether this distribution plays a direct role in filamentation, perhaps by generating a transhyphal electrical current, remains to be determined. Alternatively, these results may reflect delayed appearance of Pma1-GFP due to time required for protein synthesis and folding.

Because glucose is key to regulation of the Pma1p enzyme, we next tested whether inactivating Pma1p via glucose starvation before initiating hyphal or biofilm formation led to phenotypic differences. No difference in filamentation or biofilm formation was observed under these conditions (data not shown).

## Discussion

Cytosolic pH homeostasis is critical for the survival of the cell. However, we have found that severe cytosolic acidification is not lethal in *C. albicans*. Cells in which Pma1p activity is decreased by more than 75% have surprisingly acidic cytosols (pH ≤ 5.5) and yet exhibit minimal growth defects (Figure 2). Even wild-type cells exhibited relatively acidic cytosols (between pH 6.25 and pH 6.5, Figure 2). These results are consistent with previously published reports of whole-cell intracellular pH (pH 5.8–pH 9.0) and with reports of cytosolic pH (pH 6.0–pH 7.0) in wild-type *C. albicans* yeast cells (Cassone et al., 1983; Kaur et al., 1988; Stewart et al., 1989; Rabaste et al., 1995; Liu and Köhler, 2015; Tournu et al., 2017). Intriguingly, acidification of the cytosol affects filamentation capacity but does not prevent filamentation entirely (Figure 7). As Pma1p is likely essential in *C. albicans*, we hypothesize that these surprising results have more to do with the remarkable pH adaptability of *C. albicans* as a species than the overall importance of the Pma1p proton pump or cytosolic pH regulation to growth and filamentation.

In both *S. cerevisiae* and *C. albicans*, C-terminal truncation of Pma1p decreases Pma1p levels and ATPase activity at the plasma membrane, depressing overall growth. However, truncation is more tolerated in *C. albicans*: growth rate in an 18-aa truncation mutant decreases by over 40% in *S. cerevisiae* (Mason et al., 2014), whereas growth rate is unaffected in an 18-aa truncation mutant in *C. albicans* (Figure 2). This is despite a similar effect of 18-aa truncation on ATPase activity in isolated plasma membranes in both species, and despite decreased Pma1p levels at the plasma membrane in the *C. albicans* mutant compared to the *S. cerevisiae* mutant. These differences may be a side effect of the extraordinary pH adaptability of *C. albicans* discussed above. Still, our results confirm that the C-terminus of Pma1p in *C. albicans* is essential for proper trafficking and activity of the enzyme at the plasma membrane, much like *S. cerevisiae*.

We have observed that truncation of Pma1p mitigates the effect of both cation addition and V-ATPase inhibition on growth. These results may indicate that the growth defects associated with these treatments are caused by cytosolic acidification rather than V-ATPase or cation-specific effects. Indeed, cytosolic pH decreases upon cation addition in wild-type cells but not Pma1p truncation mutants, although we were unable to measure cytosolic pH changes upon V-ATPase inhibition due to unexpected interference of the V-ATPase inhibitor bafilomycin on pHLuorin measurements (data not shown). One possibility is that the effect of cytosolic pH acidification on growth is a universal phenomenon. If so, changes in cytosolic pH could be the underlying cause of growth defects observed upon cation addition or V-ATPase inhibition. Such growth defects occur upon genetic or chemical inhibition of V-ATPase (Bowman et al., 1988; Rane et al., 2013), and in null mutants of cation transporter genes such as *NHX1* and *VCX1* (Steinmetz et al., 2002; Orij et al., 2012; Bode et al., 2013).

Interruption of V-ATPase function leads to defects in Pma1p trafficking and activity, which in turn leads to decreased cytosolic pH (Tarsio et al., 2011; Smardon and Kane, 2014). Here we find that the inverse is not true: Pma1p truncation leads to decreased rather than increased vacuolar pH and Pma1p truncation mutants do not display the classic *vma* growth phenotype. These results indicate normal or increased V-ATPase function upon Pma1p truncation. Indeed, over-activation of V-ATPase could help compensate for loss of Pma1p activity by removing excess protons from the cytosol. Whether this type of compensation occurs is a topic of ongoing interest in our lab. Interestingly, our results clarify the role of vacuolar acidification versus vacuolar alkalinization in the secretion of degradative enzymes. Secretion of aspartyl proteases and lipases is a classically vacuole-linked phenotype, yet we observed no difference in secretion of aspartyl proteases or lipases upon Pma1p truncation despite disruption of vacuolar pH (data not shown). Vacuolar alkalinization in V-ATPase mutants, however, does lead to decreased aspartyl protease and lipase secretion (Raines et al., 2013; Rane et al., 2013). Thus, the vacuole must maintain low pH for degradative enzymes to function, however, the vacuole can be acidified well beyond wild-type levels without interrupting degradative enzyme function. These results begin to tease out the role of pH, both acidification and alkalinization, in different cell compartments during virulence.

In *S. cerevisiae*, Pma1p is inherited asymmetrically between mother and daughter cells, with mother cells retaining the majority of Pma1p (Henderson et al., 2014). The asymmetry of Pma1p inheritance in mother and daughter cells results in an asymmetry of cytosolic pH such that mother cells are more alkaline than daughter cells by ∼0.2 pH units (Henderson et al., 2014). As in *S. cerevisiae*, young daughter cells in *C. albicans* show less Pma1p than mother cells (Figure 9, time 0), whereas, older daughter cells begin to express more Pma1p. We find that Pma1p is also distributed asymmetrically in *C. albicans* hyphae (Figure 9), with Pma1p concentrations lowest proximal to the hyphal tip. Whether the asymmetry of Pma1p in *C. albicans* hyphae leads to an asymmetry of cytosolic pH along growing filaments is a topic of ongoing study in our lab. The uneven distribution of Pma1p could generate a pH gradient along the hyphae, resulting in an electric field that could drive hyphal formation (Gow, 1984). Monk et al. (1993) showed that emerging hyphae have increased ATPase-specific activity compared to yeast. In this study, we found that overall Pma1-GFP fluorescence is higher in hyphal cells than in yeast cells (Figure 9). Interestingly, the increase in Pma1p-GFP in hyphae is concentrated at the plasma membrane of the mother cell rather than along the hyphal tube. Monk et al. also examined Pma1p distribution in hyphal cells via immunofluorescence microscopy in formaldehyde-fixed cells (Monk et al., 1993). Under these conditions, Pma1p distribution was uniform in both yeast cells and germ tubes, however, formaldehyde treatment may interfere with the endogenous distribution of the enzyme *in vivo*.

Decreased Pma1p activity in the Pma1p truncation mutants impacts hyphal formation under some but not all conditions. Pma1p truncation leads to impaired filamentation in a rapid assay for filamentation in liquid media. In this assay, filamentation begins within a few minutes, and by 30 min, clear differences in Pma1p truncation mutant filamentation capacity are seen. Thus, while the presence of shorter hyphae can be due to a growth defect, the kinetics of hyphal formation in the liquid assay support a role for Pma1p in filamentation. Further, chemical inhibition of Pma1p using sub-inhibitory concentrations of ebselen also impacts hyphal formation (Figure 8). In addition, Pma1p truncation results in aberrant cell polarity. While Pma1p truncation does lead to impaired growth, taken together, these results suggest that the role of Pma1p in filamentation is not solely growth-dependent. Interestingly, overexpression of *PMA1* in a tetR-*PMA1* strain led to decreased hyphal formation on weak-inducing media (Spider, RPMI, and M199 media; data not shown). We hypothesize that overexpression of the enzyme could interfere with the distribution of Pma1p in hyphae by increasing the amount of Pma1p found along the emerging filament, thus interrupting the pH gradient generated prior to filamentation and disrupting hyphal formation. It is evident that *PMA1* expression levels must be finely tuned, as decreasing the amount of Pma1p at the plasma membrane interferes with growth and hyphal formation, yet increasing the expression of *PMA1* also interrupts hyphal formation. Taken together, our results suggest a key role for *PMA1* in regulation of filamentation; the exact parameters of that role are still under investigation.

A previous study found that expression of *C. albicans* H^+^-ATPase was unable to fully complement an H^+^-ATPase-deficient *S. cerevisiae* strain (Keniya et al., 2013), potentially indicating the evolution of functional differences between the enzymes. Interestingly, many pathogenic ascomycetes have evolved a second H^+^-ATPase, *PMA2*, that may be specialized for virulence (Fernandes et al., 2017). Our observation of non-homogeneous Pma1p distribution in hyphae and prior observations of cytosolic pH alkalinization before filamentation in *C. albicans* (Stewart et al., 1988, 1989) point to a role for *C. albicans* Pma1p in the cytosolic pH regulation of hyphal formation, a potentially newly-evolved function. Future work will continue to define that role, and unravel *Candida*-specific functions of Pma1p in pathogenesis.

The essentiality and fungal specificity of Pma1p make an attractive drug target, as previous authors have pointed out (Monk et al., 1995a,b; Perlin et al., 1997). Yet no Pma1p-specific inhibitor has been discovered to date. One reason may be that though Pma1p has no direct mammalian ortholog, the pump shares considerable structural similarity to other P-type ATPases, including mammalian H^+^/K^+^ ATPases. Thus, common proton pump inhibitors like omeprazole and rabeprazole have activity against both mammalian H^+^/K^+^ ATPases and fungal Pma1p (Monk et al., 1995a). However, the C-terminal region of the Pma1p protein is more evolutionarily diverged. The last 100 amino acids of the *C. albicans* Pma1p protein share 72% identity and 81% positivity with *S. cerevisiae*; the rest of the protein is 86% identical and 92% positive between the two species. Further, the last 200 amino acids of *C. albicans* Pma1p have no mammalian sequence similarity. Our study shows that targeting the C-terminal region of Pma1p specifically could overcome some of the limitations of Pma1p as an antifungal drug target.

## Data Availability

The datasets generated for this study are available on request to the corresponding author.

## Author Contributions

SL, KP, SH, and HR conceived and designed the experiments. HR, JF, EA, and SH performed the experiments. HR, SB, and JF analyzed the data. KP and SL contributed materials, analytical tools. HR and SL wrote the manuscript.

## Conflict of Interest Statement

The authors declare that the research was conducted in the absence of any commercial or financial relationships that could be construed as a potential conflict of interest.

## References

[B1] Ambesi A. Miranda M. Petrov V. V. Slayman C. W. (2000). Biogenesis and function of the yeast plasma-membrane H(+)-ATPase. *J. Exp. Biol.* 203 155–160. 10600684 10.1242/jeb.203.1.155

[B2] Bates S. Hughes H. B. Munro C. A. Thomas W. P. H. MacCallum D. M. Bertram G. (2006). Outer chain N-glycans are required for cell wall integrity and virulence of *Candida albicans*. *J. Biol. Chem.* 281 90–98. 10.1074/jbc.M510360200 16263704

[B3] Becker J. M. Kauffman S. J. Hauser M. Huang L. Lin M. Sillaots S. (2010). Pathway analysis of *Candida albicans* survival and virulence determinants in a murine infection model. *Proc. Natl. Acad. Sci. U.S.A.* 107 22044–22049. 10.1073/pnas.1009845107 21135205 PMC3009777

[B4] Benito B. Moreno E. Lagunas R. (1991). Half-life of the plasma membrane ATPase and its activating system in resting yeast cells. *Biochim. Biophys. Acta* 1063 265–268. 1826456 10.1016/0005-2736(91)90381-h

[B5] Bernardo S. M. Lee S. A. (2010). *Candida albicans* SUR7 contributes to secretion, biofilm formation, and macrophage killing. *BMC Microbiol.* 10:133. 10.1186/1471-2180-10-133 20433738 PMC2887802

[B6] Billack B. Santoro M. Lau-Cam C. (2009). Growth inhibitory action of ebselen on fluconazole-resistant *Candida albicans*: role of the plasma membrane H+-ATPase. *Microb. Drug Resist.* 15 77–83. 10.1089/mdr.2009.0872 19432523

[B7] Bode M. Longen S. Morgan B. Peleh V. Dick T. P. Bihlmaier K. (2013). Inaccurately assembled cytochrome c oxidase can lead to oxidative stress-induced growth arrest. *Antioxid. Redox Signal.* 18 1597–1612. 10.1089/ars.2012.4685 23198688 PMC3613174

[B8] Bowman E. J. Siebers A. Altendorf K. (1988). Bafilomycins: a class of inhibitors of membrane ATPases from microorganisms, animal cells, and plant cells. *Proc. Natl. Acad. Sci. U.S.A.* 85 7972–7976. 2973058 10.1073/pnas.85.21.7972 PMC282335

[B9] Cabezón V. Llama-Palacios A. Nombela C. Monteoliva L. Gil C. (2009). Analysis of *Candida albicans* plasma membrane proteome. *Proteomics* 9 4770–4786. 10.1002/pmic.200800988 19824013

[B10] Cassone A. Carpinelli G. Angiolella L. Maddaluno G. Podo F. (1983). 31P nuclear magnetic resonance study of growth and dimorphic transition in *Candida albicans*. *Microbiology* 129 1569–1575. 10.1099/00221287-129-5-1569 6352859

[B11] Chan G. Hardej D. Santoro M. Lau-Cam C. Billack B. (2007). Evaluation of the antimicrobial activity of ebselen: role of the yeast plasma membrane H+-ATPase. *J. Biochem. Mol. Toxicol.* 21 252–264. 10.1002/jbt.20189 17912695

[B12] Crandall M. Edwards J. E. Jr. (1987). Segregation of proteinase-negative mutants from heterozygous *Candida albicans*. *J. Gen. Microbiol.* 133 2817–2824. 3329678 10.1099/00221287-133-10-2817

[B13] Dechant R. Saad S. Ibáñez A. J. Peter M. (2014). Cytosolic pH regulates cell growth through distinct GTPases, Arf1 and Gtr1, to promote Ras/PKA and TORC1 activity. *Mol. Cell* 55 409–421. 10.1016/j.molcel.2014.06.002 25002144

[B14] Dennison P. M. J. Ramsdale M. Manson C. L. Brown A. J. P. (2005). Gene disruption in *Candida albicans* using a synthetic, codon-optimised Cre-loxP system. *Fungal Genet. Biol.* 42 737–748. 10.1016/j.fgb.2005.05.006 16043373

[B15] Diakov T. T. Tarsio M. Kane P. M. (2013). Measurement of vacuolar and cytosolic pH in vivo in yeast cell suspensions. *J. Vis. Exp.* 74:e50261. 10.3791/50261 23629151 PMC3665305

[B16] Eraso P. Gancedo C. (1987). Activation of yeast plasma membrane ATPase by acid pH during growth. *FEBS Lett.* 224 187–192. 2960558 10.1016/0014-5793(87)80445-3

[B17] Fernandes T. R. Segorbe D. Prusky D. Di Pietro A. (2017). How alkalinization drives fungal pathogenicity. *PLoS Pathog.* 13:e1006621. 10.1371/journal.ppat.1006621 29121119 PMC5679519

[B18] Fu Y. Ibrahim A. S. Fonzi W. Zhou X. Ramos C. F. Ghannoum M. A. (1997). Cloning and characterization of a gene (*LIP1*) which encodes a lipase from the pathogenic yeast *Candida albicans*. *Microbiology* 14 331–340. 9043110 10.1099/00221287-143-2-331

[B19] Gow N. A. R. (1984). Transhyphal electrical currents in fungi. *Microbiology* 130 3313–3318. 10.1099/00221287-130-12-3313 6520604

[B20] Henderson K. A. Hughes A. L. Gottschling D. E. (2014). Mother-daughter asymmetry of pH underlies aging and rejuvenation in yeast. *eLife* 3:e03504. 10.7554/eLife.03504 25190112 PMC4175738

[B21] Jin Y. Yip H. K. Samaranayake Y. H. Yau J. Y. Samaranayake L. P. (2003). Biofilm-forming ability of *Candida albicans* is unlikely to contribute to high levels of oral yeast carriage in cases of human immunodeficiency virus infection. *J. Clin. Microbiol.* 41 2961–2967. 12843027 10.1128/JCM.41.7.2961-2967.2003 PMC165379

[B22] Kaur S. Mishra P. Prasad R. (1988). Dimorphism-associated changes in intracellular pH of *Candida albicans*. *Biochim. Biophys. Acta* 972 277–282. 10.1016/0167-4889(88)90202-9 2904280

[B23] Liu H. Köhler J. Fink G. R. (1994). Suppression of hyphal formation in *Candida albicans* by mutation of a STE12 homolog. *Science* 266 1723–1726. 7992058 10.1126/science.7992058

[B24] Liu N.-N. Köhler J. R. (2015). Antagonism of fluconazole and a proton pump inhibitor against *Candida albicans*. *Antimicrob. Agents Chemother.* 60 1145–1147. 10.1128/AAC.02043-15 26596946 PMC4750695

[B25] Mason A. B. Allen K. E. Slayman C. W. (2014). C-terminal truncations of the *Saccharomyces cerevisiae* PMA1 H+-ATPase have major impacts on protein conformation, trafficking, quality control, and function. *Eukaryotic Cell* 13 43–52. 10.1128/EC.00201-13 24186948 PMC3910955

[B26] McCusker J. H. Perlin D. S. Haber J. E. (1987). Pleiotropic plasma membrane ATPase mutations of *Saccharomyces cerevisiae*. *Mol. Cell. Biol.* 7 4082–4088. 2963211 10.1128/mcb.7.11.4082-4088.1987 PMC368079

[B27] Miesenböck G. De Angelis D. A. Rothman J. E. (1998). Visualizing secretion and synaptic transmission with pH-sensitive green fluorescent proteins. *Nature* 394 192–195. 10.1038/28190 9671304

[B28] Milne S. W. Cheetham J. Lloyd D. Aves S. Bates S. (2011). Cassettes for PCR-mediated gene tagging in *Candida albicans* utilizing nourseothricin resistance. *Yeast* 28 833–841. 10.1002/yea.1910 22072586

[B29] Monk B. C. Kurtz M. B. Marrinan J. A. Perlin D. S. (1991). Cloning and characterization of the plasma membrane H(+)-ATPase from *Candida albicans*. *J. Bacteriol.* 173 6826–6836. 1834633 10.1128/jb.173.21.6826-6836.1991 PMC209034

[B30] Monk B. C. Mason A. B. Abramochkin G. Haber J. E. Seto-Young D. Perlin D. S. (1995a). The yeast plasma membrane proton pumping ATPase is a viable antifungal target. I. Effects of the cysteine-modifying reagent omeprazole. *Biochim. Biophys. Acta* 1239 81–90. 7548148 10.1016/0005-2736(95)00133-n

[B31] Monk B. C. Mason A. B. Kardos T. B. Perlin D. S. (1995b). Targeting the fungal plasma membrane proton pump. *Acta Biochim. Pol.* 42 481–496. 8852338

[B32] Monk B. C. Niimi K. Lin S. Knight A. Kardos T. B. Cannon R. D. (2005). Surface-active fungicidal D-peptide inhibitors of the plasma membrane proton pump that block azole resistance. *Antimicrob. Agents Chemother.* 49 57–70. 10.1128/AAC.49.1.57-70.2005 15616276 PMC538910

[B33] Monk B. C. Niimi M. Shepherd M. G. (1993). The *Candida albicans* plasma membrane and H(+)-ATPase during yeast growth and germ tube formation. *J. Bacteriol.* 175 5566–5574. 8366041 10.1128/jb.175.17.5566-5574.1993 PMC206613

[B34] Nailis H. Coenye T. Van Nieuwerburgh F. Deforce D. Nelis H. J. (2006). Development and evaluation of different normalization strategies for gene expression studies in *Candida albicans* biofilms by real-time PCR. *BMC Mol. Biol.* 7:25. 10.1186/1471-2199-7-25 16889665 PMC1557526

[B35] Nakayama H. Mio T. Nagahashi S. Kokado M. Arisawa M. Aoki Y. (2000). Tetracycline-regulatable system to tightly control gene expression in the pathogenic fungus *Candida albicans*. *Infect. Immun.* 68 6712–6719. 11083786 10.1128/iai.68.12.6712-6719.2000 PMC97771

[B36] Navarre C. Catty P. Leterme S. Dietrich F. S. Gouffeau A. (1994). Two distinct genes encode small isoproteolipids affecting plasma membrane H(+)-ATPase activity of *Saccharomyces cerevisiae*. *J. Biol. Chem.* 269 21262–21268. 8063750

[B37] Navarre C. Ghislain M. Leterme S. Ferroud C. Dufour J.-P. Gouffeau A. (1992). Purification and complete sequence of a small proteolipid associated with the plasma membrane H(+)-ATPase of *Saccharomyces cerevisiae*. *J. Biol. Chem.* 267 6425–6428. 1532582

[B38] Ni L. Snyder M. (2001). A genomic study of the bipolar bud site selection pattern in *Saccharomyces cerevisiae*. *Mol. Biol. Cell* 12 2147–2170. 11452010 10.1091/mbc.12.7.2147 PMC55669

[B39] Orij R. Urbanus M. L. Vizeacoumar F. J. Giaever G. Boone C. Nislow C. (2012). Genome-wide analysis of intracellular pH reveals quantitative control of cell division rate by pH(c) in *Saccharomyces cerevisiae*. *Genome Biol.* 13:R80. 10.1186/gb-2012-13-9-r80 23021432 PMC3506951

[B40] Ottilie S. Goldgof G. M. Cheung A. L. Walker J. L. Vigil E. Allen K. E. (2018). Two inhibitors of yeast plasma membrane ATPase 1 (ScPma1p): toward the development of novel antifungal therapies. *J. Chem.* 10:6. 10.1186/s13321-018-0261-3 29464421 PMC5820243

[B41] Owegi M. A. Carenbauer A. L. Wick N. M. Brown J. F. Terhune K. L. Bilbo S. A. (2005). Mutational analysis of the stator subunit E of the yeast V-ATPase. *J. Biol. Chem.* 280 18393–18402. 10.1074/jbc.M412567200 15718227

[B42] Perlin D. S. Seto-Young D. Monk B. C. (1997). The plasma membrane H(+)-ATPase of fungi: a candidate drug target? *Ann. N. Y. Acad. Sci.* 834 609–617. 9405872 10.1111/j.1749-6632.1997.tb52330.x

[B43] Petrov V. V. (2010). Point mutations in Pma1 H+-ATPase of *Saccharomyces cerevisiae*: influence on its expression and activity. *Biochem. Mosc.* 75 1055–1063. 21073429 10.1134/s000629791008016x

[B44] Portillo F. de Larrinoa I. F. Serrano R. (1989). Deletion analysis of yeast plasma membrane H + -ATPase and identification of a regulatory domain at the carboxyl-terminus. *FEBS Lett.* 247 381–385. 10.1016/0014-5793(89)81375-4 2523820

[B45] Portillo F. Eraso P. Serrano R. (1991). Analysis of the regulatory domain of yeast plasma membrane H + -ATPase by directed mutagenesis and intragenic suppression. *FEBS Lett.* 287 71–74. 10.1016/0014-5793(91)80018-X 1831768

[B46] Portillo F. Serrano R. (1988). Dissection of functional domains of the yeast proton-pumping ATPase by directed mutagenesis. *EMBO J.* 7 1793–1798. 2901955 10.1002/j.1460-2075.1988.tb03010.x PMC457170

[B47] Pringle J. R. (1991). Staining of bud scars and other cell wall chitin with calcofluor. *Meth. Enzymol.* 194 732–735. 2005820 10.1016/0076-6879(91)94055-h

[B48] Rabaste F. Sancelme M. Delort A.-M. Blais J. Bolard J. (1995). Intracellular pH of Candida albicans blastospores as measured by laser microspectrofluorimetry and 31P-NMR. *Biochim. Biophys. Acta* 1268 41–49. 10.1016/0167-4889(95)00042-Q 7626661

[B49] Raines S. M. Rane H. Bernardo S. M. Binder J. L. Lee S. A. Parra K. J. (2013). Deletion of V-ATPase Voa isoforms clarifies the role of vacuolar pH as a determinant of virulence-associated traits in *C. albicans*. *J. Biol. Chem.* 288 6190–6201. 10.1074/jbc.M112.426197 23316054 PMC3585055

[B50] Ramage G. López-Ribot J. L. (2005). Techniques for antifungal susceptibility testing of *Candida albicans* biofilms. *Methods Mol. Med.* 118 71–79. 10.1385/1-59259-943-5:071 15888936

[B51] Rane H. S. Bernardo S. M. Hayek S. R. Binder J. L. Parra K. J. Lee S. A. (2014). The contribution of *Candida albicans* vacuolar ATPase subunit V1B, encoded by *VMA2*, to stress response, autophagy, and virulence is independent of environmental pH. *Eukaryot. Cell* 13 1207–1221. 10.1128/EC.00135-14 25038082 PMC4187624

[B52] Rane H. S. Bernardo S. M. Raines S. M. Binder J. L. Parra K. J. Lee S. A. (2013). *Candida albicans* VMA3 is necessary for V-ATPase assembly and function and contributes to secretion and filamentation. *Eukaryot. Cell* 12 1369–1382. 10.1128/EC.00118-13 23913543 PMC3811332

[B53] Rao R. Slayman C. W. (1996). “Plasma membrane and related ATPases,” in *The Mycota*, eds Brambl R. Marzluf G. (Berlin: Springer-Verlag), 29–56.

[B54] Schindelin J. Arganda-Carreras I. Frise E. Kaynig V. Longair M. Pietzsch T. (2012). Fiji: an open-source platform for biological-image analysis. *Nat. Methods* 9 676–682. 10.1038/nmeth.2019 22743772 PMC3855844

[B55] Segal E. S. Gritsenko V. Levitan A. Yadav B. Dror N. Steenwyk J. L. (2018). Gene essentiality analyzed by *in vivo* transposon mutagenesis and machine learning in a stable haploid isolate of *Candida albicans*. *mBio* 9:e02048–18. 10.1128/mBio.02048-18 30377286 PMC6212825

[B56] Serrano R. (1983). In vivo glucose activation of the yeast plasma membrane ATPase. *FEBS Lett.* 156 11–14. 6221943 10.1016/0014-5793(83)80237-3

[B57] Serrano R. Kielland-Brandt M. C. Fink G. R. (1986). Yeast plasma membrane ATPase is essential for growth and has homology with (Na+ + K+), K+- and Ca2+-ATPases. *Nature* 319 689–693. 10.1038/319689a0 3005867

[B58] Skrzypek M. S. Binkley J. Binkley G. Miyasato S. R. Simison M. Sherlock G. (2017). The *Candida* Genome Database (CGD): incorporation of Assembly 22, systematic identifiers and visualization of high throughput sequencing data. *Nucleic Acids Res.* 45 D592–D596. 10.1093/nar/gkw924 27738138 PMC5210628

[B59] Smardon A. M. Kane P. M. (2014). Loss of vacuolar H+-ATPase activity in organelles signals ubiquitination and endocytosis of the yeast plasma membrane proton pump Pma1p. *J. Biol. Chem.* 289 32316–32326. 10.1074/jbc.M114.574442 25271159 PMC4231704

[B60] Steinmetz L. M. Scharfe C. Deutschbauer A. M. Mokranjac D. Herman Z. S. Jones T. (2002). Systematic screen for human disease genes in yeast. *Nat. Genet.* 31 400–404. 10.1038/ng929 12134146

[B61] Stewart E. Gow N. A. Bowen D. V. (1988). Cytoplasmic alkalinization during germ tube formation in *Candida albicans*. *J. Gen. Microbiol.* 134 1079–1087. 3058860 10.1099/00221287-134-5-1079

[B62] Stewart E. Hawser S. Gow N. A. (1989). Changes in internal and external pH accompanying growth of *Candida albicans*: studies of non-dimorphic variants. *Arch. Microbiol.* 151 149–153. 2655548 10.1007/BF00414430

[B63] Tarsio M. Zheng H. Smardon A. M. Martínez-Muñoz G. A. Kane P. M. (2011). Consequences of loss of Vph1 protein-containing vacuolar ATPases (V-ATPases) for overall cellular pH homeostasis. *J. Biol. Chem.* 286 28089–28096. 10.1074/jbc.M111.251363 21669878 PMC3151054

[B64] Tournu H. Luna-Tapia A. Peters B. M. Palmer G. E. (2017). In vivo indicators of cytoplasmic, vacuolar, and extracellular pH using pHluorin2 in *Candida albicans*. *mSphere* 2:e0276–17. 10.1128/mSphere.00276-17 28685162 PMC5497024

[B65] Weibull W. (1951). A statistical distribution function of wide applicability. *J. Appl. Mechan.* 18 293–297.

[B66] Wilson R. B. Davis D. Enloe B. M. Mitchell A. P. (2000). A recyclable *Candida albicans URA3* cassette for PCR product-directed gene disruptions. *Yeast* 16 65–70. 10620776 10.1002/(SICI)1097-0061(20000115)16:1<65::AID-YEA508>3.0.CO;2-M

