# Supplementary material for: Candida albicans Pma1p Contributes to Growth, pH Homeostasis, and Hyphal Formation
Source: Front Microbiol. 2019 May 9;10:1012. doi: 10.3389/fmicb.2019.01012 (PMC6521590; doi:10.3389/fmicb.2019.01012)
Supplement: Supplementary file 1 [file Table_1.docx]

**Supplemental Table 1.** List of strains used in this study.

| Strain | Parent | Relevant genotype | Source |
| --- | --- | --- | --- |
| BWP17 | SC5314 | *ura3*Δ*/ura3*Δ *arg4*Δ*/arg4*Δ *his*Δ*/his1*Δ *PMA1/PMA1* | Wilson et al. 1999 |
| THE1 | CAI8 | *ade2*Δ::*hisG/ade2*Δ::*hisG ura3*Δ::*imm434/ura3*Δ::*imm434* *ENO1/eno1*Δ::*ENO1*-tetR-ScHAP4AD-3xHA-*ADE2 PMA1/PMA1* | Nakayama et al. 2000 |
| THE1-CIp10 | THE1 | *ade2*Δ::*hisG*/*ade2*Δ::*hisG ura3*Δ::*imm434*/*ura3*Δ::*imm434* *ENO1*/*eno1*Δ::*ENO1*-tetR-ScHAP4AD-3xHA-*ADE2 RP10/RP10*::*URA3 PMA1/PMA1* | Bernardo et al. 2008 |
| tetR – *PMA1* | THE1 | *ura3*Δ::*imm434*/*ura3*Δ::*imm434 PMA1*Δ::*dpl200*::99t-*PMA1*-*URA3 ade2*Δ::*hisG*/*ade2*Δ::*hisG ura3*Δ::*imm434*/*ura3*Δ::*imm434* *ENO1*/*eno1*Δ::*ENO1*-tetR-ScHAP4AD-3xHA-*ADE2* | This study |
| *pma1*Δ/+ | BWP17 | *ura3*Δ*/ura3*Δ *arg4*Δ*/arg4*Δ *his*Δ*/his1*Δ *PMA1*/*PMA1*Δ::*dpl200*-*URA3*-*dpl200* | This study |
| *pma1*Δ/+-CIp30 | *pma1*Δ+ | *ura3*Δ*/ura3*Δ *arg4*Δ*/arg4*Δ *his*Δ*/his1*Δ *PMA1*/*PMA1*Δ::*dpl200 RP10/RP10*::*URA3::HIS::ARG4* | This study |
| Δ878 | *pma1*Δ/+ | *ura3*Δ*/ura3*Δ *arg4*Δ*/arg4*Δ *his*Δ*/his1*Δ *PMA1*Δ::*dpl200/PMA1*Δ878*::ARG4 RP10/RP10*::*URA3::HIS* | This study |
| Δ866 | *pma1*Δ/+ | *ura3*Δ*/ura3*Δ *arg4*Δ*/arg4*Δ *his*Δ*/his1*Δ *PMA1*Δ::*dpl200/PMA1*Δ866*::ARG4 RP10/RP10*::*URA3::HIS* | This study |
| Δ864 | *pma1*Δ/+ | *ura3*Δ*/ura3*Δ *arg4*Δ*/arg4*Δ *his*Δ*/his1*Δ *PMA1*Δ::*dpl200/PMA1*Δ864*::ARG4 RP10/RP10*::*URA3::HIS* | This study |
| Δ862 | *pma1*Δ/+ | *ura3*Δ*/ura3*Δ *arg4*Δ*/arg4*Δ *his*Δ*/his1*Δ *PMA1*Δ::*dpl200/PMA1*Δ862*::ARG4 RP10/RP10*::*URA3::HIS* | This study |
| Δ858 | *pma1*Δ/+ | *ura3*Δ*/ura3*Δ *arg4*Δ*/arg4*Δ *his*Δ*/his1*Δ *PMA1*Δ::*dpl200/PMA1*Δ858*::ARG4 RP10/RP10*::*URA3::HIS* | This study |
| Δ878+pHLuorin | Δ878 | *ura3*Δ*/ura3*Δ *arg4*Δ*/arg4*Δ *his*Δ*/his1*Δ *PMA1*Δ::*dpl200/PMA1*Δ878*::ARG4 RP10/RP10*::*URA3::HIS P_ACT1_-ACT1/P_ACT1_-pHLuorin-ACT1* | This study |
| Δ866+pHLuorin | Δ866 | *ura3*Δ*/ura3*Δ *arg4*Δ*/arg4*Δ *his*Δ*/his1*Δ *PMA1*Δ::*dpl200/PMA1*Δ866*::ARG4 RP10/RP10*::*URA3::HIS P_ACT1_-ACT1/P_ACT1_-pHLuorin-ACT1* | This study |
| Δ864+pHLuorin | Δ864 | *ura3*Δ*/ura3*Δ *arg4*Δ*/arg4*Δ *his*Δ*/his1*Δ *PMA1*Δ::*dpl200/PMA1*Δ864*::ARG4 RP10/RP10*::*URA3::HIS P_ACT1_-ACT1/P_ACT1_-pHLuorin-ACT1* | This study |
| Δ862+pHLuorin | Δ862 | *ura3*Δ*/ura3*Δ *arg4*Δ*/arg4*Δ *his*Δ*/his1*Δ *PMA1*Δ::*dpl200/PMA1*Δ862*::ARG4 RP10/RP10*::*URA3::HIS P_ACT1_-ACT1/P_ACT1_-pHLuorin-ACT1* | This study |
| Δ858+pHLuorin | Δ858 | *ura3*Δ*/ura3*Δ *arg4*Δ*/arg4*Δ *his*Δ*/his1*Δ *PMA1*Δ::*dpl200/PMA1*Δ858*::ARG4 RP10/RP10*::*URA3::HIS P_ACT1_-ACT1/P_ACT1_-pHLuorin-ACT1* | This study |
| *PMA1-*GFP | SC5314 | *PMA1/PMA1-GFP* | This study |
