# Supplementary material for: Candida albicans Pma1p Contributes to Growth, pH Homeostasis, and Hyphal Formation
Source: Front Microbiol. 2019 May 9;10:1012. doi: 10.3389/fmicb.2019.01012 (PMC6521590; doi:10.3389/fmicb.2019.01012)
Supplement: Supplementary file 2 [file Table_2.docx]

**Supplemental Table 2.** Primers used in this study.

| Primer | Primer sequence (5’ – 3’) | Source |
| --- | --- | --- |
| PMA1-5DR | ACTTTTTTTTTTCAATCTTTGTTTTTGGTTAATTAATCTTAAGAATAAGGGATTTTTATATATATATAAACCGTTTTCCCAGTCACGACGTT | This study |
| PMA1-3DR | AAATTAGTAATAACAATTGTCAATTAATATTAATCTCCAATTATTCACACCAACAACGATATCATCAAATTGTGGAATTGTGAGCGGATA | This study |
| PMA1-5Det | CGCTCGCTCGCATTTTCTCA | This study |
| PMA1-3Det | CTGGGAAAGGAATCAGCAAT | This study |
| tetPMA1-5DR | ATTGCAAAGTCAAAACTATATAAGTACCATTACAATTCCCATCAAATAATTCATATCAATTGTATTTCCAGTAATACGACTCACTATAGGG | This study |
| tetPMA1-3DR | CAATGTCTTCGTCTTCATCATCGGAGACGATTTTATCAACCTTTTCGTTGGTTGGTTCAGTAGCACTCATCTAGTTTTCTGAGATAAAGCTG | This study |
| tetPMA1-3Det | CTTCTTCATCACCAGCACCTG | This study |
| PMP1-5DR | TTCTTTTCCTTTTCTTCTTTCAATTGAAATATTTCAAATCAATAATTCCAATAATAAATACCAAACCATAGTTTTCCCAGTCACGACGTT | This study |
| PMP1-3DR | GGATAGTTAACTTATGATAAAACTATCTAGACTTTGGAATAGCATTATCAATTGCTATTTAATTGTCTTTTGTGGAATTGTGAGCGGATA | This study |
| PMP1-5Det | TCAAGAAGAAGAGAAGTCATG | This study |
| PMP1-3Det | GGATTGACAATTATTCCCCA | This study |
| ∆878p-5DR | TACTTCCGAAGCCTTTGACAACTTCTGTAACGGTAGAAAACCACAACAACACACTGACAAGAGATCCTTGTAAGTTTTCCCAGTCACGACGTT | This study |
| ∆866p-5DR | CTTCTGTGTCATGGGTGGTGCTTACTACTTGATGTCTACTTCCGAAGCCTTTGACAACTTCTGTAACGGTTAAGTTTTCCCAGTCACGACGTT | This study |
| ∆864p-5DR | CGGTGTCTTCTGTGTCATGGGTGGTGCTTACTACTTGATGTCTACTTCCGAAGCCTTTGACAACTTCTGTTAAGTTTTCCCAGTCACGACGTT | This study |
| ∆862p-5DR | GTCTTTCGGTGTCTTCTGTGTCATGGGTGGTGCTTACTACTTGATGTCTACTTCCGAAGCCTTTGACAACTAAGTTTTCCCAGTCACGACGTT | This study |
| ∆858p-5DR | AACCTGGATTTGGTCTTTCGGTGTCTTCTGTGTCATGGGTGGTGCTTACTACTTGATGTCTACTTCCGAATAAGTTTTCCCAGTCACGACGTT | This study |
| ∆878p-3DR | ACAAACATGCAAAATTAGTAATAACAATTGTCAATTAATATTAATCTCCAATTATTCACACCAACAACGATGTGGAATTGTGAGCGGATA | This study |
| PMA1-qRT-5 | TTGCTTATGATAATGCTCCATACGA | Nailis et al., 2006 |
| PMA1-AMP-5 | GGTTTGTCCGGTGGTGGTGAC | This study |
| PMA1-AMP-3 | CCATATGAAAGAACGGAGGATG | This study |
| PMA1-SEQ-5 | CCGTATTGCCCTTTCATTGC | This study |
| PMA1-SEQ-3 | GAGATGCTCTTGGTGGTACTGC | This study |
| PMA1-GFP-5DR | TGACAAGAGATCCTTGGAAGATTTCCTTGTGTCCATGCAAAGAGTATCTACTCAACACGAAAAATCTACTGGTGGTGGTTCTAAAGGTGAAGAATTATT | This study |
| PMA1-GFP-3DR | AAACATGCAAAATTAGTAATAACAATTGTCAATTAATATTAATCTCCAATTATTCACACCAACAACGATACGTTAGTATCGAATCGACAGC | This study |
| PMA1-RT-5 | GAAGTCTTGAACGGTATTGG | This study |
| FJK1514 | GACACCTAACTAATAAACC | Liu and Köhler, 2015 |
| RJK1515 | CCAGTAAATAATTCTTCACC | Liu and Köhler, 2015 |
| FJK1517 | GGAATTGTGAGCGGATAAC | Liu and Köhler, 2015 |
| RJK1516 | CAATCAATTAGAATTGAAGC | Liu and Köhler, 2015 |
