# Supplementary material for: Candida albicans Pma1p Contributes to Growth, pH Homeostasis, and Hyphal Formation
Source: Front Microbiol. 2019 May 9;10:1012. doi: 10.3389/fmicb.2019.01012 (PMC6521590; doi:10.3389/fmicb.2019.01012)
Supplement: Supplementary file 3 [file Data_Sheet_1.PDF]

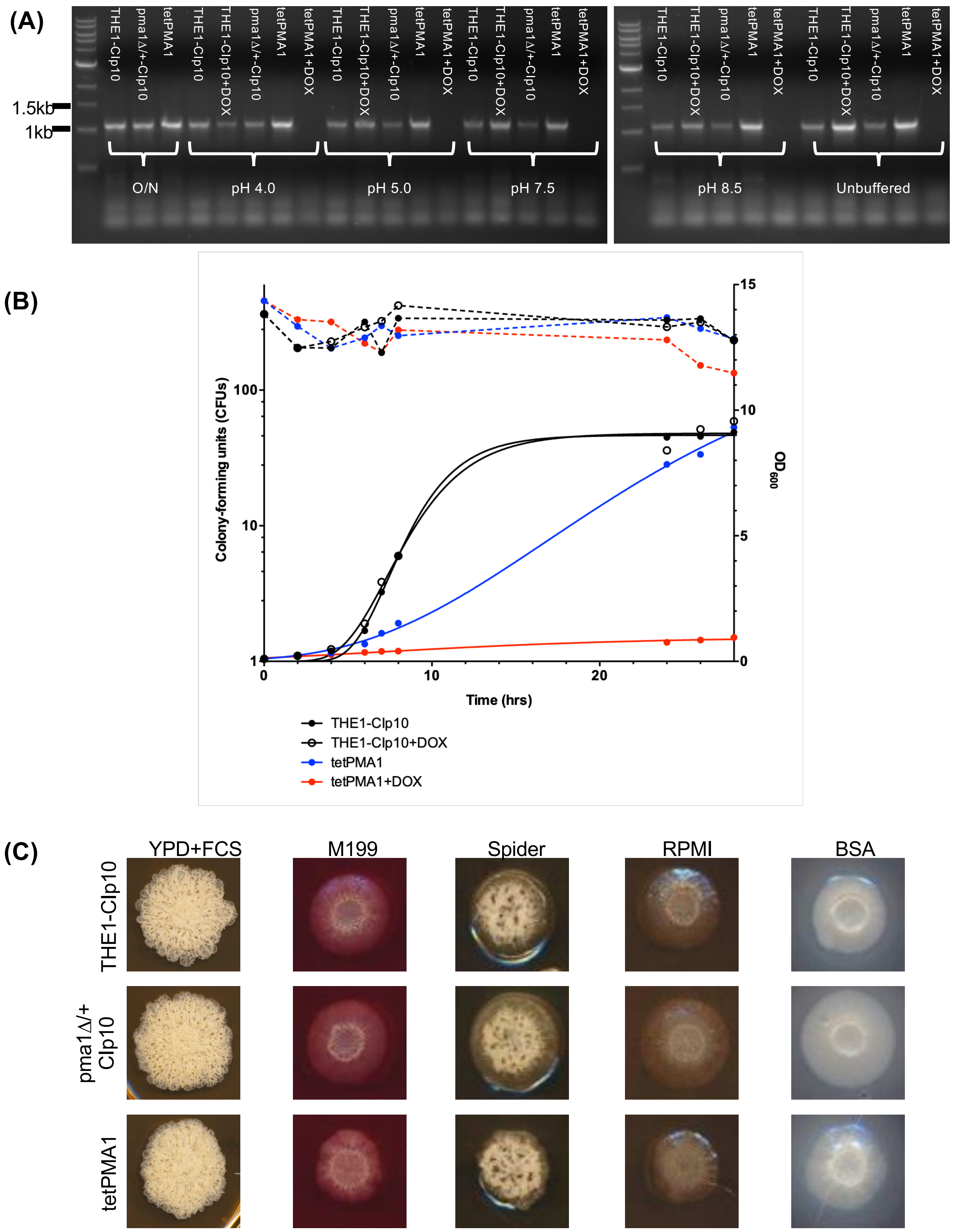

Supplemental figure 1. Characterization of a tetracycline-regulatable mutant, tetPMA1. (A) RT-PCR of *PMA1* levels in tetPMA1. RNA was extracted from saturated cultures grown overnight in YPD (i.e., O/N), or from cells grown for 24h in synthetic dropout media buffered to pH 4, 5, 7.5, 8.5 or without a pH buffer added (unbuffered, pH ~6.0), with and without doxycycline (DOX). RT-PCR was completed using primers inside the *PMA1* open reading frame. *PMA1* expression is increased in the tetPMA1 strain in the absence of doxycycline, but absent upon addition of 20µg/µl DOX. (B) Growth and viability of tetPMA1. Growth was assessed in YPD in the presence and absence of DOX by measuring OD_{600} at fixed intervals, after strains were diluted to a starting OD_{600} of 0.1. Viability was assessed via colony forming units, determined at the indicated time points by plating a fixed number of cells on YPD agar medium. In the presence of DOX, tetPMA1 is viable but exhibits little to no growth. Overepxression of *PMA1* in the tetPMA1 strain without DOX impacts growth but not viability. (C) Filamentation on agar plates without doxycycline. 3 µL OD-corrected cells from overnight cultures were spotted onto YPD+FCS, M199, RPMI and Spider agar plates and incubated at 37°C for 5 days. Overexpression of *PMA1* in the tetPMA1 strain leads to decreased filamentation on weak-inducing media (M199, Spider and RPMI).
